# Discovery of a new marker to identify myeloid cells associated with metastatic breast tumours

**DOI:** 10.1186/s12935-023-03136-w

**Published:** 2023-11-18

**Authors:** Ansooya A. Bokil, Mathieu Le Boulvais Børkja, Camilla Wolowczyk, Apsana Lamsal, Wenche S. Prestvik, Unni Nonstad, Kristine Pettersen, Sonja B. Andersen, Anna M. Bofin, Geir Bjørkøy, Sjoerd Hak, Miriam S. Giambelluca

**Affiliations:** 1 Department of Circulation and Medical Imaging, Faculty of Medicine and Health Sciences, Norwegian University of Science and Technology, Trondheim, Norway; 2 Centre of Molecular Inflammation Research (CEMIR), Faculty of Medicine and Health Sciences, Norwegian University of Science and Technology, Trondheim, Norway; 3 Department of Clinical and Molecular Medicine, Faculty of Medicine and Health Sciences, Norwegian University of Science and Technology, Trondheim, Norway; 4 Department of Biomedical Laboratory Science, Faculty of Natural Sciences, Norwegian University of Science and Technology, Trondheim, Norway; 5 Department of Biotechnology and Nanomedicine, SINTEF, Trondheim, Norway; 6 Department of Clinical Medicine, Faculty of Health Science, UiT- The Arctic University of Norway, Tromsø, Norway

**Keywords:** Metastatic tumours, Myeloid-derived cells, Arginase 1, Prognostic marker

## Abstract

**Background:**

Myeloid cells play an essential role in cancer metastasis. The phenotypic diversity of these cells during cancer development has attracted great interest; however, their functional heterogeneity and plasticity have limited their role as prognostic markers and therapeutic targets.

**Methods:**

To identify markers associated with myeloid cells in metastatic tumours, we compared transcriptomic data from immune cells sorted from metastatic and non-metastatic mammary tumours grown in BALB/cJ mice. To assess the translational relevance of our in vivo findings, we assessed human breast cancer biopsies and evaluated the association between arginase 1 protein expression in breast cancer tissues with tumour characteristics and patient outcomes.

**Results:**

Among the differentially expressed genes, arginase 1 (ARG1) showed a unique expression pattern in tumour-infiltrating myeloid cells that correlated with the metastatic capacity of the tumour. Even though ARG1-positive cells were found almost exclusively inside the metastatic tumour, ARG1 protein was also present in the plasma. In human breast cancer biopsies, the presence of ARG1-positive cells was strongly correlated with high-grade proliferating tumours, poor prognosis, and low survival.

**Conclusion:**

Our findings highlight the potential use of ARG1-positive myeloid cells as an independent prognostic marker to evaluate the risk of metastasis in breast cancer patients.

**Graphical Abstract:**

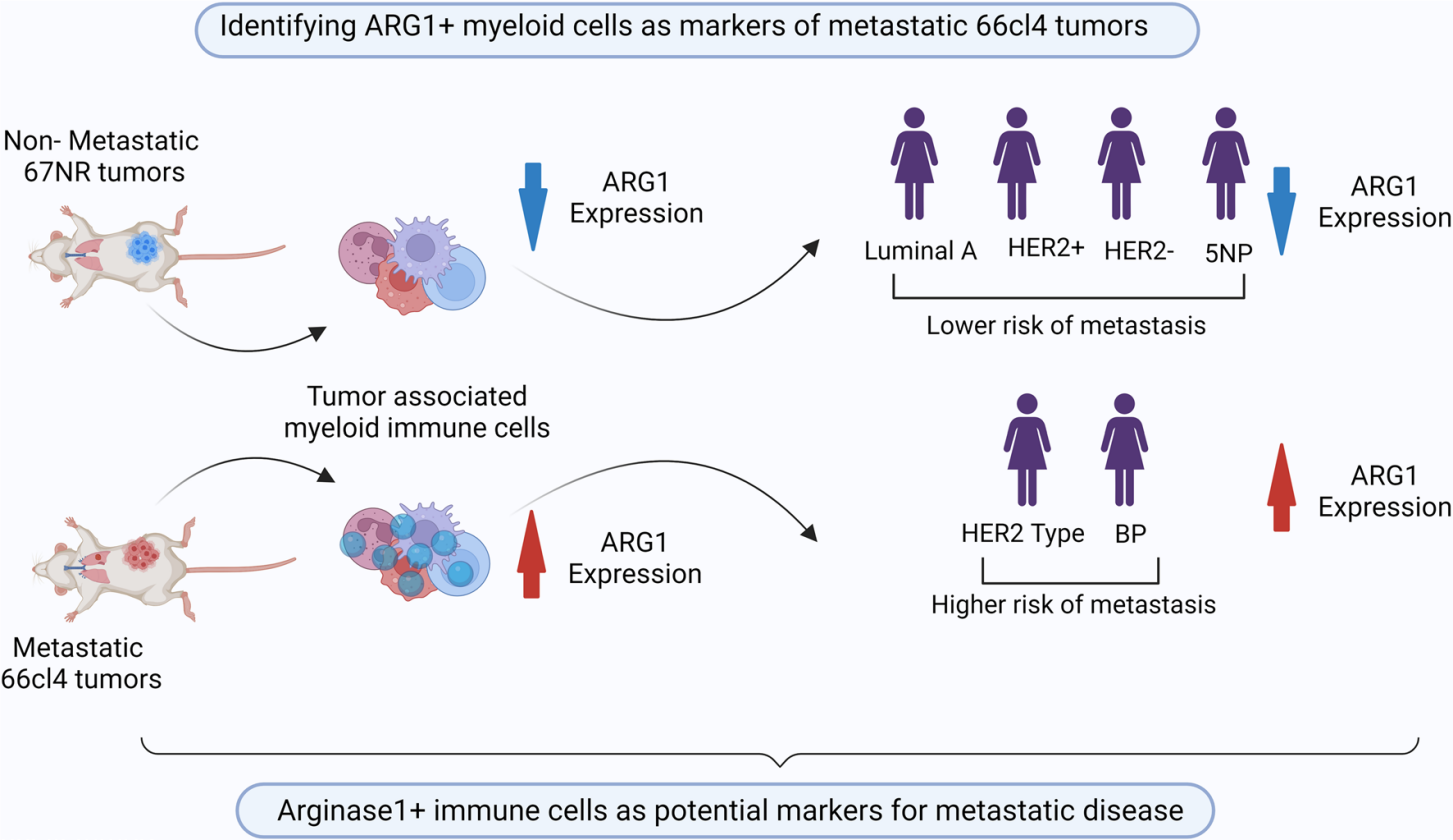

**Supplementary Information:**

The online version contains supplementary material available at 10.1186/s12935-023-03136-w.

## Background

Despite exceptional advances in anti-cancer therapy, cancer metastases continue to be the leading cause of cancer-related mortality [1]. Advancements in our understanding of cancer metastasis have highlighted the crucial role of tumour microenvironment (TME), particularly immune cells. These cells are implicated at each step of tumour development and thus influential in its progression [2]. To establish primary tumours, cancer cells must not only escape immune surveillance, but also modulate immune cell behaviour [3, 4]. Due to their extensive involvement, several therapeutic strategies have been developed to enhance the anti-tumour activity of immune cells. However, few of these so-called immunotherapeutic strategies have entered the clinic. Most approved immunotherapies are based on immune checkpoint blockade (ICB) or adoptive T cell transfer that strengthens anti-tumour T cell responses [5, 6]. Although highly successful in certain cancers and individuals, the response rate of patients receiving mono-immunotherapy remains modest at approximately 20% [7]. This low response rate can be attributed to an immunosuppressed TME and aberrant communication between the innate and adaptive immune systems.

It is well established that myeloid cells have various roles as mediators in tumour development and metastasis [8]. Pro-tumourigenic and pro-metastatic activities of myeloid cells include suppression of adaptive immune responses [9], promotion of angiogenesis, matrix remodelling, and preparation of the pre-metastatic niche [10, 11]. Moreover, increased numbers of myeloid cells within solid tumours [12, 13] and a high neutrophil to lymphocyte ratio in blood, are associated with poor prognosis and unfavourable clinical outcomes in patients with certain types of tumours [13], confirming a pro-metastatic role of myeloid cells. Although these prognostic approaches are currently being studied, the correlation between the number of tumour infiltrating myeloid cells and patient survival requires more insights given the heterogenous nature of these cells [14, 15]. It is therefore imperative to further our understanding of myeloid cell biology in the context of cancer if we aim to use myeloid cells as prognostic markers and therapeutic targets in the clinic.

Here, we compared immune cell transcriptomes in primary metastatic versus non-metastatic tumours originating from two cell lines derived from the mammary carcinoma model 4T1. We utilized the cell lines 67NR, which forms non-metastatic tumours, and 66cl4, which forms tumours that metastasize to the lungs [16]. Our results showed significant differences in the immune cell functions associated with these two tumour types and revealed several molecular candidates that could potentially serve as prognostic markers. Arginase 1 (ARG1) gene and protein was highly expressed in myeloid cells of metastatic tumours, while it was nearly absent in these cells originating from non-metastatic ones. Even though ARG1 expression was induced upon immune cell infiltration into the tumour, a small number of ARG1-positive myeloid cells were also found in the lungs, which is the main metastasis site for 66cl4 tumours. Interestingly, ARG1 protein levels in the metastatic tumour also correlated with its levels and activity in plasma. This highlights ARG1 plasma abundance as a potential marker for the presence of pro-metastatic myeloid cells in the TME. Finally, we performed immunohistochemistry on 487 human breast cancer biopsies and found that the number of ARG1-positive cells correlated with high-grade proliferating tumours, poor prognosis, and low survival. In conclusion, this study indicates that ARG1-positive myeloid cells harbour potential as an independent prognostic factor in breast cancer.

## Materials and methods

### Mouse model

Animal experiments were approved by the National Animal Research Authorities, Norwegian Food and Safety Authority (FOTS: 17895 and FOTS: 26021) and carried out according to the European Convention for the Protection of Vertebrates used for Scientific Purposes. Eight- to twelve-week-old female BALB/cJ mice were purchased from Janvier Labs. France, and housed (3–6 mice/cage) under controlled light/temperature/humidity conditions with ad libitum access to food and water. Tumour growth and mouse weight were monitored and followed the pattern we reported previously [17]. The sample size for each experiment was decided based on our previous experience with these tumours [17, 18].

### Cell culture and tumour induction

The 67NR and 66cl4 cell lines were obtained from the Karmanos Cancer Institute, Detroit, MI, USA. The cells were cultured in Dulbecco’s modified Eagle’s medium (Lonza, BioWhittaker, Cat #BE12-604F) supplemented with 10% fetal calf serum (FCS, Thermo Fisher Scientific, Gibco, Cat #10272-106), 2 mM L-glutamine (Lonza, Cat #BEBP17-605E), and 50 U/mL penicillin–streptomycin (Thermo Fischer Scientific, Gibco, Cat #15070063). Cells were incubated at 37°C in 5% CO_2_ and allowed to grow to 75% confluence, after which they were used to prepare a cell suspension of 20 × 10^6^ cells/mL in sterile PBS (Sigma-Aldrich, Cat #D8537). For orthotopic tumour induction, mice were selected randomly and 1 × 10^6^ viable cells were injected into the inguinal (fourth) mammary fat pad of mice under anesthesia (1.5% isoflurane with 67.5% N_2_ and 32.5% O_2_). Tumour growth and mouse weight were monitored weekly, and the experiments were performed 21–26 days after injection. Control mice were healthy mice without injections. The total number mice used: controls N = 21; 67NR N = 25; and 66cl4 N = 25; none of the mice showed unexpected or adverse effects.

### Tissue processing

Blood was collected from mice under anaesthesia (4% isoflurane with 67.5% N_2_ and 32.5% O_2_) by cardiac puncture in heparin-containing tubes. Then, it was centrifuged at 1500 × *g* for 10 min, and plasma was collected and stored at −80°C until analysis (ELISA and LC–MS/MS). If the collected plasma showed haemolysis, it was excluded from the analysis. Leukocytes were obtained after red cell lysis using RBC lysis buffer (Thermofisher Scientific, 1X RBC Lysis Buffer, Cat #00-4333-57).

After blood collection, mice were euthanised by cervical dislocation and organs were harvested. Bone marrow cells were obtained by flushing the marrow cavities of the femurs with PBS using a syringe with a 26-gauge needle, followed by red cell lysis using RBC lysis buffer. Splenic cells were obtained by gently dissociating small spleen pieces through a 70 μm sterile cell strainer. Tumour and lung single-cell suspensions were prepared by mincing 0.5 mm pieces in an uncoated Petri dish containing 2 mL of serum-free RPMI using a scalpel. The minced tissue was placed in a tube containing a mix of Liberase DL (0.835 U/mL, Roche, Cat #5466202001), Liberase TL (0.835 U/mL, Roche, Cat #5401020001) and DNase I (13 U/mL, Qiagen, Cat #79254) in DMEM for 30 min at 37°C on a shaking incubator. The tissue single-cell suspensions were washed with serum-free RPMI, and one red-cell lysis step was performed. Samples were centrifuged at 400 × *g* for 8 min, and single cells were suspended in cold FACS buffer (PBS, supplemented with 2% fetal calf serum and 2 mM EDTA). Tissues showing > 50% necrosis were excluded.

### Flow cytometry

The single cell suspensions obtained by tissue processing explained above were resuspended in FACS buffer and incubated with 0.5 μg of Fc block antibody (anti-mouse CD16/32, Affymetrix, eBioscience, Cat #14-0161-85) for 15 min on ice. Surface staining of cell suspensions was performed by addition of anti-mouse CD45-FITC (Biolegend, Cat #103108, 0.5 μg/test), CD11b-BV421 (Biolegend, Cat #101251, 1 μg/test) and Ly6G-PE/Cy7 (Biolegend, Cat #127618, 0.5 μg/test) antibodies and incubation for 20 min on ice protected from light. After incubation, the cell suspensions were washed twice, and samples were stained with Zombie Aqua (BioLegend, Cat #423101) for 10 min on ice, according to the manufacturer’s instructions. After surface staining, cells were washed twice with cold FACS buffer and fixed using the Fixation Buffer (R&D Systems, Cat #FC009) for 10 min at RT according to the manufacturer’s instructions. Following fixation, the cells were washed twice and resuspended in 200 μL of Permeabilization Buffer (R&D Systems, Cat #FC009) to perform the intracellular staining. The cells were incubated with 0.5 μg of Fc block antibody (anti-mouse CD16/32) for 15 min, followed by 30 min with ARG1-APC antibody (Invitrogen, Cat #17-3697-82, 1 μg/test) at RT. Finally, the cells were washed twice, resuspended in FACS buffer and analysed on a BD LSR II flow cytometer.

Myeloid cell subpopulations were identified by surface staining with CD45-FITC, CD11b-BV421, Ly6G-PE/Cy7, Ly6C-PerCP/Cy5.5 (Biolegend, Cat #128012, 0.5 μg/test), F4/80-PE-Texas Red (ThermoScientific, Cat #MF48017, 1 μg/test) and ARG1-APC antibodies.

Single staining and fluorescence-minus-one (FMO) staining were performed. Antibody volumes were used according to manufacturer recommendations. The generated data was analysed using FlowJo software v10. Gating strategies for the analyses are described in supplementary data.

### Cell sorting

The single-cell suspensions obtained by tissue processing explained above were resuspended in FACS buffer and incubated with 0.5 μg of Fc block antibody (anti-mouse CD16/32) for 15 min on ice. Then, the cells were incubated with CD45-FITC antibody for 30 min on ice, followed by staining with the viability dye Zombie Aqua, as mentioned above. Cells were washed, resuspended in FACS buffer and sorted based on CD45 expression levels using a BD FACSAria™ Fusion flow cytometer.

### RNA sequencing

RNA sequencing was performed on the RNA extracted from the sorted populations. RNA quantity, quality, integrity, and purity were evaluated using Qubit, bioanalyser and NanoDrop. Sequencing libraries were generated using the SENSE mRNA-Seq library prep kit V2, according to the manufacturer’s instructions (Lexogen GmbH), with 350 ng of total RNA as input.

Libraries were normalised to 2.6 pM, subjected to clustering and single-read sequencing. According to the manufacturer's instructions, sequencing was performed for 86 cycles on a NextSeq500 HO flow cell (Illumina). Base calling was performed using NextSeq500 instrument software (RTA 2.4.6). FASTQ files were generated using the bcl2fastq2 Conversion Software v2.20.0.422. The sequences are available from February 01, 2024 under the GEO NCBI Accession code: GSE211223.

### RNA sequencing data analysis

FASTQ files were quality controlled with fastqc (v0.11.9), filtered and trimmed using fastp (v0.20.0). Trimmed sequences were aligned to the reference genome using STAR (v2.7.3), and quality metrics were extracted with Picard CollectRNASeqMetrics (v2.21.5). Transcript counts were generated using quasi-alignment (Salmon v1.3.0) to GRCh38 transcriptome reference sequences. Transcript counts were imported into R statistical software and aggregated to gene counts using the tximport (v1.14.0) Bioconductor package for downstream statistical analysis. Gene counts were normalised and analysed for differential expression using the DESeq2 Bioconductor package. Differential expression was defined as genes with a false discovery adjusted p-value < 0.05 and log fold change difference > 1. Pathway enrichment analysis was performed using the enriched Bioconductor (v3.0) package.

### Quantitative real-time PCR

Total RNA from the whole tumour was obtained by first placing small tumour pieces in lysis buffer (RLT, provided in RNeasy^®^ Mini Quiagen Kit, Cat #74106) and then disrupted with 2.8 mm ceramic beads using an MP Biomedical FastPrep-24™ tissue homogeniser (SKU 116004500), with two rounds of 4.0 m/s for 20 s each. Samples were vortexed, and RNA was isolated following manufacturer instructions (RNeasy® Mini Kit, Qiagen, Cat #74106). Total RNA from sorted CD45^+^ and CD45^-^ cells was obtained using the Quick-RNA™ MicroPrep Kit (Zymo Research, Cat #R1051), following manufacturer instructions. RNA was quantified by spectrophotometry (ND1000 Spectrophotometer, NanoDrop, Thermo Scientific) and stored at −80˚C until further analysis. cDNA was synthesised from 500 ng (whole tumour) and 100 ng (sorted cells) total RNA using a High-Capacity cDNA Reverse Transcription Kit (Applied Biosystems™, Cat #4387406). Quantitative real-time PCR (RT-qPCR) was performed in 20 µl reactions containing 10 µl of 2X QuantiTect SYBR Green PCR master mix (Qiagen), 2 µl 10X QuantiTect Primer Assay and 8 µl of the sample containing 2 ng of cDNA. QuantiTect Primers for Mm_Hprt_1_SG (Cat #QT00166768) -as a reference gene- and Mm_Arg_1_SG (Cat #QT00134288) were used. RT-qPCR was performed on the StepOne Plus system (Applied Biosystems) using the following cycling conditions: 95°C for 10 min, followed by 40 cycles at 95°C for 15 s, 60°C for 30 s and 72°C for 30 s. Relative gene expression levels were calculated using the 2^(-ΔΔCT)^ method.

### Immunoblotting

Primary tumour pieces were homogenised in lysis buffer containing 8 M urea, 4% CHAPS (Cat #C9426), 1 M DTT (Cat #646563), Complete® protease inhibitor (Cat #1187350001) and phosphatase inhibitor cocktail II and III (Cat #P5726 and #P0044). Homogenised tissues were placed on a shaker for 20 min at 4°C and centrifuged at 16,000 × *g* for 20 min at 4°C. Protein concentration was measured using BioRad protein assay (Cat #5000006). Equal amounts of proteins were separated using NuPAGE® Novex® Bis–Tris gels (4–12%) (Invitrogen), transferred onto nitrocellulose membranes and stained with Revert™ 700 Total Protein Stain (P/N: 926–11016). After destaining and washing, the membrane was further stained with ARG1 (GeneTex, Cat #GTX109242, 1:2000) and ERK1/2 (Cell Signaling, Cat #9107S, 1:2000) antibodies. Near-infrared fluorescent secondary antibodies were used and imaged on an Odyssey Near Infrared Scanner (Li-Cor Biosciences). Images were processed using Li-Cor Odyssey software Image Studio v5.2.5.

### Fluorescence in situ hybridisation (FISH)

Formalin-fixed paraffin-embedded 66cl4 and 67NR tumours were cut into 4 µm sections using a microtome, mounted on Superfrost Plus slides, dried and used for FISH.

The RNAScope Pretreatment Kit (ACD Bio, Cat #310020) was used according to the manufacturer’s instructions. Following pretreatment, the sections were stained using the RNAScope 2.5 Detection Kit- Brown (ACD Bio, Cat #322310) and washed with RNAscope Wash Buffer (ACD Bio, Cat #310091) following the manufacturer’s instructions. The probe Mm-ARG1 (ACD Bio, Cat #403431) was used. Images were captured using an IX71 inverted fluorescence microscope (Olympus).

### Enzyme-linked immunosorbent assay (ELISA)

Levels of ARG 1 protein in plasma samples, obtained from tumour-bearing and healthy mice, were determined using a Mouse Arginase 1 ELISA kit (Abcam, Cat #ab269541). Only samples with no visual evidence of haemolysis were analyzed. Plasma samples were diluted at 1:40, analyzed according to the manufacturer’s protocol and absorbance at 450 nm was read on the Bio-Rad iMark Microplate Reader Bio-Rad (Cat #168-1135).

### Immunohistochemistry (IHC)

Formalin-fixed paraffin-embedded 66cl4 and 67NR tumours were cut into 4 µm sections using a microtome, mounted on Superfrost Plus slides and dried. Antigen retrieval was performed using citrate buffer (pH 6; Cat #C9999). Slide preparation and staining were performed using a Rabbit-specific HRP/DAB (ABC) Detection IHC Kit (Cat #ab64261) and VECTASTAIN Elite ABC-HRP Kit, Peroxidase (Rat IgG) (PK-6104), respectively, following the manufacturer’s instructions for all steps except treatment after primary staining. After primary staining, the slides were treated with hydrogen peroxide, and the manufacturer’s protocol was resumed. The slides were incubated with an anti-ARG1 antibody (Cell Signaling, Cat #93668, 1:100) for 1 h at room temperature.

Images were captured using an IX71 inverted fluorescence microscope (Olympus).

### LC–MS/MS

Metabolites were extracted, measured and analysed using a previously described method [19].

### Statistics

GraphPad Prism 9.2.0 (332) was used for statistical analysis. For the analysis of 2 groups, we used Mann–Whitney t-tests (Unpaired) and Kruskal–Wallis test (Unpaired) for more than two groups, followed by a multiple comparisons test.

### Data availability

The data are scheduled to be publicly available from February 01, 2024, in the Gene Expression Omnibus (GEO) at GSE211223. The data are available earlier upon request.

## Human samples

### Breast cancer subtypes cohort 1

The human breast cancer tissue used in this study was obtained from a cohort of 25,727 women born between 1886 and 1928, who participated in a population-based early breast cancer detection program in Nord-Trøndelag, Norway, between 1956 and 1959 [20]. A total of 1396 patients diagnosed with breast cancer during the follow-up period from 1961 to 2008 and 909 of them, were reclassified into molecular subtypes [21] and followed up from the time of diagnosis until death from breast cancer, death from other causes, or the 31st of December 2015.

Use of human samples was approved by the Regional Committee for Medical and Health Research Ethics in Central Norway (Approval number: REK 836-09). This study was approved by the general requirements for patient consent and performed in accordance with the Declaration of Helsinki.

### Specimen characteristics

Each tumour was previously classified into histopathological type and grade according to established guidelines [22, 23]. Then, three 1 mm tissue cores were extracted from the periphery of each primary tumour and assembled in tissue microarrays (TMA) using a Tissue Arrayer MiniCore with TMA Designer2 software (Alphelys). These were subsequently cut into 4 μm thick sections and kept frozen at −20°C until use. As previously described, the 909 tumours were classified into the following molecular subtypes: luminal A (ER^+^ and/or PR^+^, HER2^−^, Ki67 < 15%), luminal B (HER2^−^) (ER^+^ and/or PR^+^, HER2^−^, Ki67 ≥ 15%), luminal B (HER2^+^) (ER^+^ and/or PR^+^, HER2^+^), HER2 type (ER^−^, PR^−^, HER2^+^), five negative phenotype (5NP) (ER^−^, PR^−^, HER2^-^, cytokeratin 5 (CK5)^−^, epidermal growth factor receptor 1 (EGFR)^−^, and basal phenotype (BP) (ER^−^, PR^−^, HER2^−^, CK5^+^, and/or EGFR^+^)[21]. The present study restricted itself to 521 primary tumours from a more recent date (from the 1980s onwards), of which 34 were excluded due to insufficient tissue, leaving a final number of 487 cases for analysis.

### Immunohistochemistry (IHC)

For ARG1 IHC, the Dako EnVison + Dual Link System-HRP (DAB+) (Cat #K4065) was used, followed by the procedure described above for IHC of the mouse breast cancer model.

### Scoring and reporting

ARG1-stained human breast cancer tissues were assessed by bright-field microscopy. The number of positively stained infiltrating polymorphonuclear cells within a single TMA core was recorded in each case. Where more than one well-preserved tissue core was available, the core with the highest number of positively stained cells was chosen. If no apparent differences were observed, one was chosen arbitrarily. Cells were recorded as infiltrating if they were located within the tumour, either in the epithelial compartment or stromal compartment, but not visibly contained in the vessels. Subsequently, cases were divided into two categories: < 10 and ≥ 10 ARG1 positively stained cells.

### Statistics

Pearson’s χ^2^ test was used to assess the association between ARG1 protein expression in human breast cancer tissues and different patient and tumour characteristics. A log-normal, accelerated failure time model was chosen for the survival analysis because the assumption of proportional hazards was inappropriate for the included covariates. The model was fitted using several different distributions (log-logistic, log-normal, Weibull, exponential, and generalised gamma), and the goodness of fit was assessed by calculating and comparing the Akaike and Bayesian information criteria for each distribution. In the final model, the impact of the included variables on survival was expressed as a time ratio with a 95% confidence interval.

## Results

### mRNA sequencing analysis of CD45^+^ and CD45^−^ cells from metastatic and non-metastatic tumours revealed distinct immune cell biological processes

To identify the main functions of immune cells in metastatic and non-metastatic tumours, we analysed the transcriptomes of 66cl4 and 67NR primary tumours. mRNA-sequencing was performed on tumour cells sorted into two populations: immune (CD45^+^) and non-immune (CD45^−^) cells. Principal component analysis (PCA) of the transcriptomes demonstrated a high degree of similarity between the five biological replicates of each tumour model (Additional file 1: Fig. S1A). Additionally, samples were separated by tumour type (67NR vs 66cl4) and cell type (immune vs non-immune), indicating differential gene expression between the tumour and cell types. Compared with 67NR CD45^+^ cells, we found that 2822 genes were differentially expressed in 66cl4 CD45^+^ cells (Log2Fold change > 1, < −1; adjusted p-value < 0.05), where 1064 genes were upregulated, and 1758 were downregulated (Additional file 1: Fig. S1B).

**Fig. 1:**
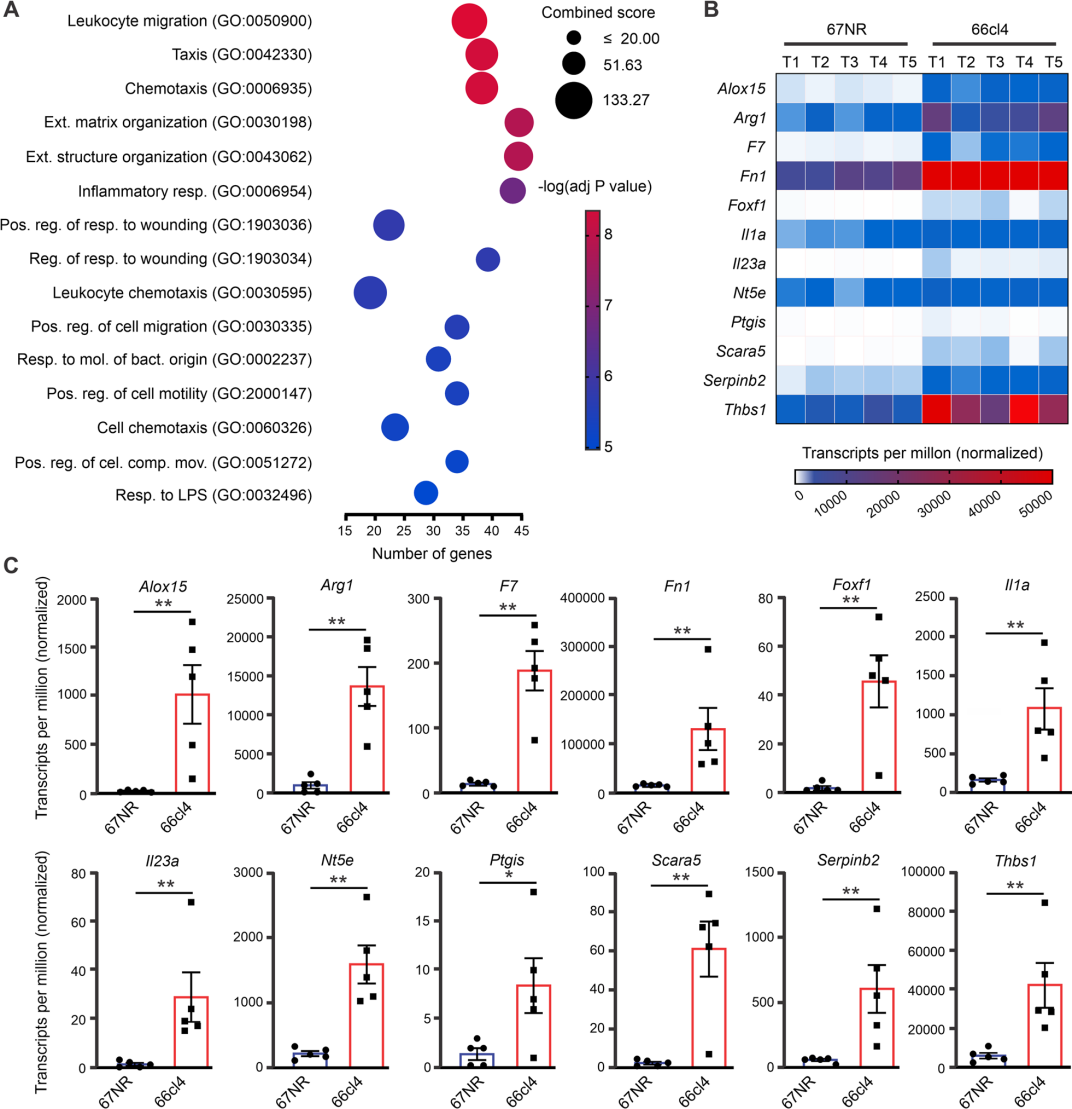
Comparative analysis of biological processes in metastatic and non-metastatic tumours**.** **A** Gene Ontology analysis shows the 15 most enriched biological processes of upregulated genes in 66cl4 CD45^+^ cells vs 67NR CD45^+^ cells. **B** Heatmap shows transcripts per million of the top 12 highest upregulated genes associated with inflammatory and wound healing responses (N = 5) in CD45^+^ cells. **C** Average expression levels of the selected genes as transcripts per million (TPM). Bars represent means ± SEM and each data point represents a single animal. Statistical significance was determined using Mann Whitney t-test, (*p < 0.05; **p < 0.005; ***p < 0.0005)

To identify the predominant pathways and biological processes where the differentially expressed 66cl4 CD45^+^ genes were involved, we performed a Gene Ontology (GO) analysis of the 500 most upregulated genes based on Log2Fold change (Additional file 1: Fig. S1C, D and Table S1). Enrichment analysis of biological processes indicated elevated expression of genes related to leukocyte migration, chemotaxis, and inflammatory and wound healing responses (Fig. 1A and Additional file 1: Table S1).

Since leukocyte migration and chemotaxis have been extensively studied [24], we focused on the genes associated with inflammatory and wound healing responses. Based on the Log2Fold change, we identified the 12 most differentially expressed genes of the 66cl4 CD45^+^ cells: *Il23a, Alox15, Scara5, Foxf1, F7, Ptgis, Thbs1, Il1a *(inflammatory and immunomodulatory functions), *Arg1* and *Nt5e*, (immunosuppressive activities) and *Fn1* and *Serpinb2,* (markers for specific immune cell subsets) (Fig. 1B). Of these, we selected the three genes with the highest transcript levels in 66cl4 CD45^+^ compared with CD45^+^ from 67NR tumours: *Arg1, Fn1* and *Thbs1* (Fig. 1C). *Arg*1 was the only gene that solely marked immune cells, whereas *Fn1* and *Thbs* were equally expressed in CD45^+^ and CD45^−^ cells (Additional file 1: Table S2). Arginase 1 (ARG1) catalyses the hydrolysis of l-arginine and decreases l-arginine levels in the TME [25]. l-arginine is essential for lymphocyte activation, and its depletion suppresses anti-tumour responses in these cells [25]. Although the immunosuppressive role of ARG1 is well established, its role as a marker of pro-metastatic immune cells, with its differential gene or protein expression in metastatic vs non-metastatic tumours, has not been extensively studied. Therefore, to explore the use of ARG1 as a pro-metastatic marker and go beyond its immunosuppressive role, we characterised the *Arg1* gene and ARG1 protein expression, as well as its cellular source and activity in metastatic and non-metastatic tumours.

### ARG1 gene and protein are differentially expressed in metastatic and non-metastatic tumours

**Fig. 2:**
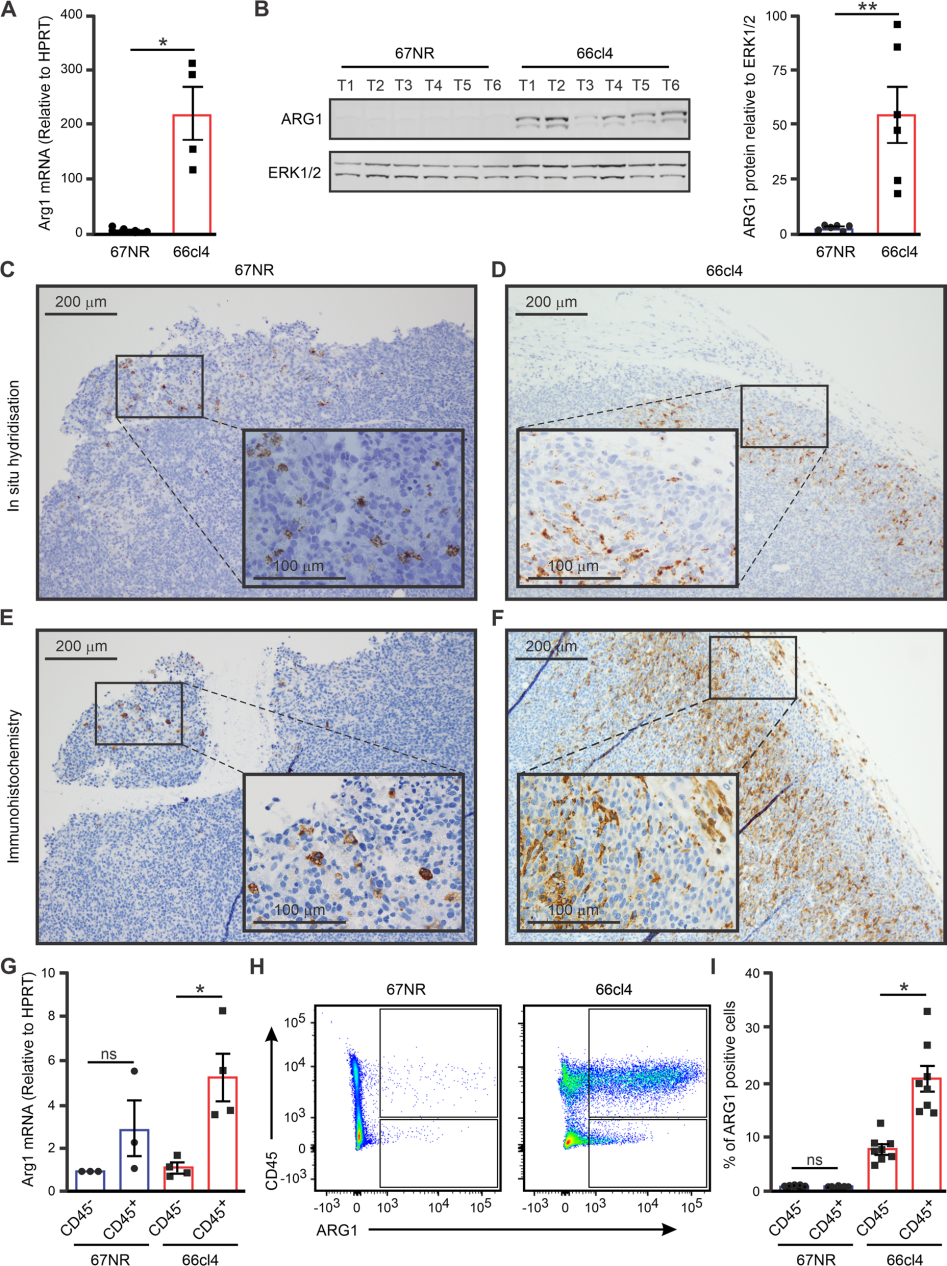
Arginase 1 gene and protein expression in the whole tumour and single cells**.** This figure shows that arginase mRNA and protein were expressed at much higher levels in metastatic than non-metastatic tumours. **A** *Arg1* mRNA levels in 67NR (N = 5) and 66cl4 (N = 5) tumours were measured using qRT-PCR. **B** Immunoblots of the ARG1 protein and ERK1/2 (loading control) in 67NR (N = 6) and 66cl4 (N = 6) tumours. **C**–**F** Representative images of in situ hybridization (**C**, **D**) and immunohistochemistry (**E**, **F**) showing the spatial distribution of *Arg1* mRNA and ARG1 protein in 67NR and 66cl4 tumours. The panels show representative images at 100X magnification, and the inset shows images at 400X magnification. **G**–**I** *Arg1* mRNA and ARG1 protein levels were significantly higher in CD45^+^ immune cells than in CD45^−^ non-immune cells from 66cl4 tumours than in CD45^+^ and CD45^−^ cells from 67NR tumours. **G** *Arg1* mRNA level in CD45^+^ and CD45^−^ from 67NR (N = 4) and 66cl4 (N = 4) tumours. **H** Representative flow cytometry plots showing CD45 vs ARG1 protein in single cells from 66cl4 and 67NR tumours. **I** Percentage of ARG1 positive CD45^+^ and CD45^−^ cells in 67NR (N = 7) and 66cl4 (N = 8) tumours. Values are presented as mean ± SEM and each data point represents a single animal. Statistical significance was determined using the Mann–Whitney test (*p < 0.05, **p < 0.005)

Using qRT-PCR and western blotting of tumour lysates, we confirmed elevated *Arg1* mRNA and ARG1 protein levels in metastatic 66cl4 tumours compared to those in non-metastatic tumours (Fig. 2A, B). To evaluate the spatial distribution of *Arg1* mRNA and ARG1 protein, we performed in situ hybridisation and immunohistochemistry on paraffin-embedded tumour tissue sections. Figure 2C, D and Additional file 1: Fig. S2A, B show representative images of *Arg1* mRNA-containing cell distribution in 67NR and 66cl4 tumours. In line with the RNA sequencing-based data, *Arg1* mRNA-containing cells were abundant in the 66cl4 tumours. Strikingly, positive cells were typically localised in the tumour periphery. The few *Arg1* mRNA-positive cells we observed in 67NR tumours, were also localised in the periphery (Fig. 2C, D and Additional file 1: Fig. S2A, B). Consistently, ARG1 protein-positive cells were more abundant in 66cl4 tumours. Although these cells displayed a similar tumour peripheral distribution as *Arg1* mRNA-containing cells (Fig. 2E, F and Additional file 1: Fig. S2C, D), the ARG1 protein-containing cells also infiltrated deeper into the tumour core (Fig. 2D, F and Additional file 1: Fig. S2D, F). At the cellular level, ARG1 protein appeared to be cytoplasmic and homogeneously distributed around the nucleus (Fig. 2E, F and Additional file 1: Fig. S2E, F). The ARG1 protein-positive cells infiltrating the 66cl4 tumour were relatively large and displayed an elongated or stellate shape with long cytoplasmic extensions (Additional file 1: Fig. S2E, F). Conversely, ARG1-positive cells in 67NR were smaller and had a round morphology. Therefore, the ARG1-containing cells in the two tumours appear to be different types of cells and may indicate an additional role for this protein in these tumours.

ARG1-containing cells show a heterogeneous morphology, indicating that ARG1 can be expressed in different cell types, such as immune cells (macrophages, neutrophils, and other myeloid cells) or non-immune cells (fibroblasts and endothelial cells). Therefore, we assessed ARG1 protein expression in sorted CD45^+^ and CD45^−^ cells from 67NR and 66cl4 tumours. Consistent with the mRNA-based results (Fig. 2G), ARG1 protein expression was associated with CD45^+^ cells and was significantly elevated in 66cl4 tumours (Fig. 2H, I and Additional file 1: Fig. S3A). These results suggest that metastatic mouse breast primary tumours contain significantly higher levels of Arg1 mRNA and ARG1 protein-containing immune cells than non-metastatic ones. Hence, these cells may be a potential marker for an immune phenotype associated with metastatic tumours.

### ARG1-positive immune cells are mainly CD11^+^/Ly6G intermediate myeloid cells

**Fig. 3:**
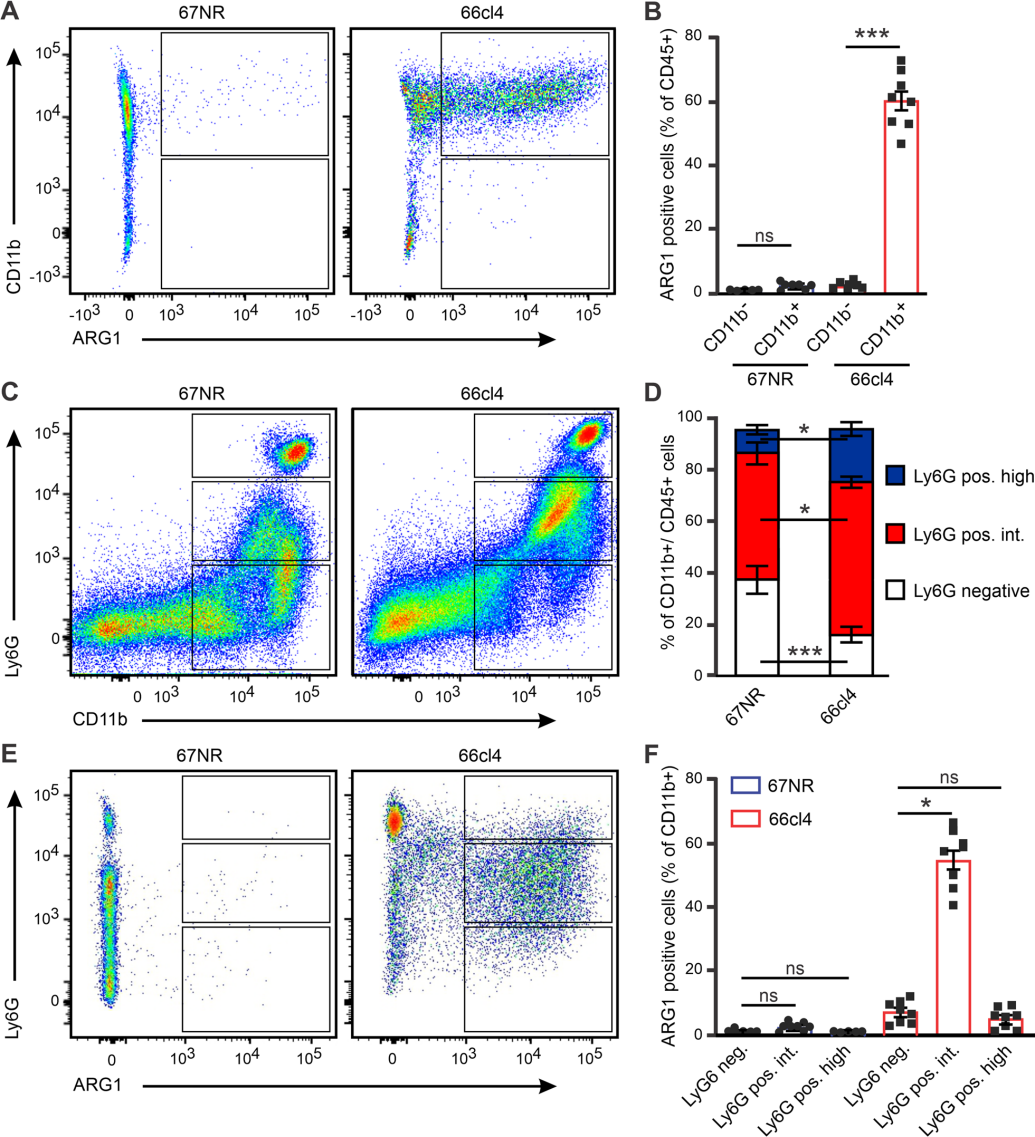
ARG1-positive myeloid cells are mainly a Ly6G intermediate population. **A** Representative flow cytometry plot to visualize ARG1 vs myeloid marker CD11b, in single cells suspension. **B** Flow cytometry-based quantification of the percentage of ARG1-positive in CD11b^+^ and CD11b^−^ cells (67NR N = 7, 66cl4 N = 8). **C** Representative flow cytometry plot to visualize the differential frequencies of CD11b vs Ly6G cells. **D** Flow cytometry-based quantification of the percentages of Ly6G negative, Ly6G positive intermediate and Ly6G positive high cells in the CD11b^+^ subset in 67NR (N = 11) and 66cl4 (N = 13) tumours. **E** Representative flow cytometry plot to visualize ARG1 vs Ly6G. **F** Flow cytometry-based quantification as ARG1-positive  cells (% of CD11b^+^ cells) in the three Ly6G populations (67NR N = 8, 66cl4 N = 10)

In metastatic 66cl4 tumours, ARG1 is predominantly expressed by infiltrating immune cells. To further identify the ARG1-positive immune cells, surface marker CD11b was stained to distinguish between myeloid (CD11b^+^) and lymphoid (CD11b^−^) cells. In both the tumours, lymphoid cells contributed to 1.5% of all ARG1-positive cells and expressed relatively low levels of ARG1. This suggested that lymphoid cells were not the primary source of ARG1. In contrast, 60% of myeloid cells in 66cl4 tumours and less than 2% in 67NR tumours expressed high levels of ARG1 (Fig. 3A, B and Additional file 1: Fig. S3B), indicating that myeloid cells are the main source of ARG1 in metastatic tumours.

Next, we used Ly6G surface expression, in CD11b^+^ cells, to differentiate neutrophils (CD11b^+^/Ly6G^+^) from monocytes/tissue-resident macrophages (CD11b^+^/Ly6G^−^) [26]. As expected, we identified three populations based on Ly6G expression: mature neutrophils with high Ly6G expression levels; presumably immature neutrophils, and other myeloid cells with intermediate Ly6G expression; and monocytes or tissue-derived macrophages with no Ly6G expression (Fig. 3C, D and Additional file 1: Fig. S4). CD45^+^/CD11b^+^ cells in 67NR tumours were mainly composed of Ly6G^−^ cells (monocytes/tissue macrophages) with a smaller fraction being Ly6G^+^ intermediate, while in 66cl4, the largest portion of CD45^+^/CD11b^+^ cells were Ly6G^+^ intermediate with a smaller fraction being Ly6G^+^ high (mature neutrophils).

To assess which cell types were present in the Ly6G negative and intermediate subsets, we used Ly6C and F4/80 surface markers to differentiate infiltrating from tissue-resident macrophages (Additional file 1: Fig. S5). In both tumours, the largest portion of the Ly6G^−^ subset was composed of Ly6C^−^/F4/80^−^ cells (myeloid-derived cells). The Ly6G^+^ intermediate subset was markedly different between the two tumours. In 67NR tumours, this subset was composed of Ly6C^−^ /F4/80^+^ cells (tissue-resident macrophages), with a smaller fraction being Ly6C^−^ F4/80^−^ cells. In 66cl4 tumours, this subset contained a significantly higher level of Ly6C^−^ F4/80^-^ cells and only half the amount of L6C^−^ F4/80^+^ cells as compared to 67NR tumours. Ly6G^+^ high cells were predominantly mature neutrophils in both tumours.

Next, we assessed ARG1 expression in the different Ly6G subsets. In 67NR tumours, only 3% of the Ly6G^+^ intermediate and negative subsets, and less than 1% of Ly6G^+^ high cells were ARG1 positive (Fig. 3E, F and Additional file 1: Fig. S6). In contrast, 40–60% of Ly6G^+^ intermediate cells and 5–11% of Ly6G^−^ and Ly6G^+^ high cells were positive for ARG1 in 66cl4 tumours, suggesting that ARG1 is a marker for a subpopulation of myeloid cells with intermediate Ly6G expression.

### ARG1-positive myeloid cells are found mainly inside the tumours

Previous reports have shown that tumour development is associated with an increase in immature and immuno-suppressive myeloid cells in the bone marrow (BM) and peripheral lymphoid organs of both cancer patients and tumour-bearing mice [27]. These suppressive cells are recruited to the tumour, where they dampen the anti-tumour response, or may migrate to sites of metastasis, where they help establish metastatic niches [28]. Since we found high levels of myeloid cells containing ARG1 in the metastatic tumour, we analysed the cells isolated from the BM, spleen, blood, and lungs for the presence of these cells. Flow cytometry of single-cell suspensions was used to score CD11b^+^/Ly6G^+^ intermediate and ARG1 positive cells from different organs of control and tumour-bearing mice. Given the difference in infiltrating myeloid cells between 67NR and 66cl4 tumours, it was surprising to find no significant difference in the frequency of CD11b^+^/Ly6G^+ ^intermediate or other myeloid cells in any organ (Additional file 1: Fig. S7A). Next, we analysed the presence of ARG1 positive myeloid cells. The only significant finding was a two-fold increase in the level of ARG1 containing myeloid cells in the lungs of 66cl4 tumour-bearing mice (Additional file 1: Fig. S7B and Table S3).

### ARG1 activity is increased in tumour and plasma samples from metastatic tumour-bearing mice

**Fig. 4:**
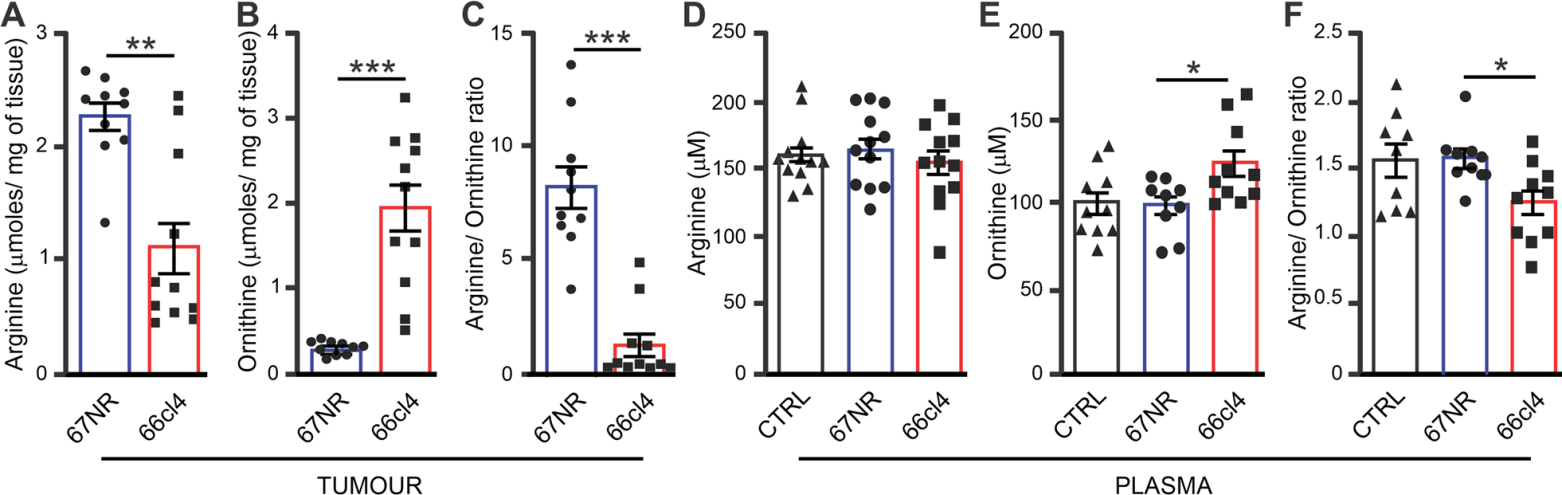
ARG1 activity in tumours and plasma. **A**–**C** Graphical representation of arginine (**A**) and ornithine (**B**) levels in tumour in µmol/mg of tissue, as measured by mass spectrometry. (**C**) The ratio of arginine to ornithine. Bars represent the mean ± SEM, and each data point represents a single tumour (67NR N = 10, 66cl4 N = 11). Statistical significance was determined using the Mann–Whitney test. *p < 0.05, **p < 0.005, ***p < 0.0005. **D**–**F** show the levels of arginine (**D**) and ornithine (**E**) in µM in plasma, while (**F**) shows the ratio of arginine to ornithine in plasma. Bars represent mean ± SEM and each data point represents a single animal (control N = 12, 67NR N = 12, 66cl4 N = 12). Statistical significance was determined using the Mann–Whitney test. *p < 0.05, **p < 0.005, ***p < 0.0005

Our results demonstrate that *Arg1* mRNA and ARG1 protein expression predominantly occurs in myeloid cells with Ly6G^-^ and Ly6G^+^ intermediate expression associated with metastasis-able 66cl4 primary tumours. Besides being a marker of immune cells, elevated ARG1 levels may have functional consequences in the TME [25]. The enzymatic function of ARG1 is to catalyse the hydrolysis of l-arginine to L-ornithine and urea. Therefore, we performed mass spectrometry analysis to determine arginine and ornithine levels in metastatic and non-metastatic tumour samples. Strikingly, arginine levels were significantly lower (Fig. 4A), and ornithine levels were markedly higher in primary tumours formed by 66cl4 cells than in 67NR cells (Fig. 4B). The low arginine:ornithine ratio suggests elevated ARG1 enzymatic activity in 66cl4 tumours (Fig. 4C).

In humans, the ARG1 protein is enriched in the azurophilic granules of neutrophils, which are released during cell activation [29]. We hypothesised that in our mouse model, ARG1 could also be released from tumour-infiltrating immune cells and leaked into the plasma, affecting arginine levels systemically. To test our hypothesis, we quantified the ARG1 protein levels in plasma from healthy and tumour-bearing mice using ELISA. ARG1 protein levels were significantly higher in the plasma of mice bearing 66cl4 tumours compared to plasma from healthy and 67NR tumour-bearing mice (Additional file 1: Fig. S8). In contrast to the intra-tumoural levels of arginine, the plasma levels of this amino acid were similar in healthy and tumour-bearing mice (Fig. 4D). However, ornithine levels were higher in plasma from 66cl4 mice than in the plasma from 67NR tumour-bearing mice (Fig. 4E). The resulting decrease in the arginine to ornithine ratio in the plasma of 66cl4 tumour-bearing mice (Fig. 4F) suggested an increase in arginase activity in the plasma of 66cl4 tumour-bearing mice.

### Significant associations of ARG1-positive cells with clinic pathological factors in human breast cancer

To assess the translational value of our findings from mice to humans, we performed IHC for ARG1 in 487 primary breast cancer tumours, including luminal A, luminal B (HER2^−^), luminal B (HER2^+^), HER2 type, 5NP, and the basal phenotype (BP). Table 1 shows the patient and tumour characteristics of the study population. The mean age at diagnosis was 75.8 years, and the mean follow-up time after diagnosis was 9.1 years. By the end of follow-up, 166 (34%) women had died from breast cancer, and 274 (56%) had died from other causes.

**Table 1:** Study population characteristics according to presence of  ≥ 10 infiltrating ARG1-positive cells per 1 mm tissue core.

	Study population	Infiltration of ≥ 10 ARG1-positive cells		
		No	Yes	p-value (χ^2^)
N (%)	487	454 (93)	33 (7)	
Mean age at diagnosis, years (SD)	75.8 (8.5)	75.9 (8.5)	74.6 (9.1)	
Mean follow-up after diagnosis, years (SD)	9.1 (7.0)	9.2 (7.0)	8.3 (8.1)	
Deaths from BC (%)	166 (34)	152 (33)	14 (42)	
Deaths from other causes (%)	274 (56)	260 (57)	14 (42)	
Grade (%)				
I	54 (11)	52 (11)	2 (6)	0.005
II	277 (57)	265 (59)	12 (36)	
III	155 (32)	136 (30)	19 (58)	
Lymph node metastasis (%)				
Yes	167 (34)	154 (34)	13 (39)	0.2
No	218 (45)	208 (46)	10 (30)	
Unknown histology	102 (21)	92 (20)	10 (30)	
Molecular type (%)				
Luminal A	259 (53)	247 (55)	12 (36)	< 0.001
Luminal B (HER2^−^)	115 (24)	11 (24)	5 (15)	
Luminal B (HER2^+^)	36 (7)	34 (8)	2 (6)	
HER2 type	27 (6)	21 (5)	6 (18)	
5NP	12 (2)	11 (2)	1 (3)	
BP	36 (7)	29 (6)	7 (21)	
Histologic subtype (%)				
Ductal (NOS)	341 (70)	321 (71)	20 (61)	0.03
Lobular	63 (13)	61 (13)	2 (6)	
Other	83 (17)	72 (16)	11 (33)	
ER status (%)				
< 1	70 (14)	58 (13)	12 (36)	0.004
≥ 1, < 10	17 (3)	15 (3)	2 (6)	
≥ 10, < 50	22 (5)	20 (4)	2 (6)	
≥ 50, < *9*0	63 (13)	58 (13)	5 (15)	
≥ 90	313 (64)	301 (66)	12 (36)	
Unknown	2 (0)	2 (0)	0 (0)	
Ki67 high/low (%)				
Ki67 < 15%	293 (60)	280 (62)	13 (39)	0.04
Ki67 ≥ 15%	192 (39)	172 (38)	20 (61)	
Mitoses/10 HPF, median (IQR p25, p75)	5 (1, 12)	5 (1, 1)	12 (4, 20)	
Mitoses/10 HPF, quartiles (%)				
≤ 1 cm	128 (26)	123 (27)	5 (15)	< 0.001
> 1, ≤ 5	131 (27)	126 (28)	5 (15)	
> 5, ≤ 12	117 (24)	107 (24)	10 (30)	
> 12	111 (23)	98 (22)	13 (39)	

N, number of patients; SD, standard deviation; BC, breast cancer; ER, estrogen receptor; HER2, human epidermal growth factor receptor 2; 5NP, 5 negative phenotypes; BP, basal phenotype; HPF, high-power field; IQR, interquartile range

**Fig. 5:**
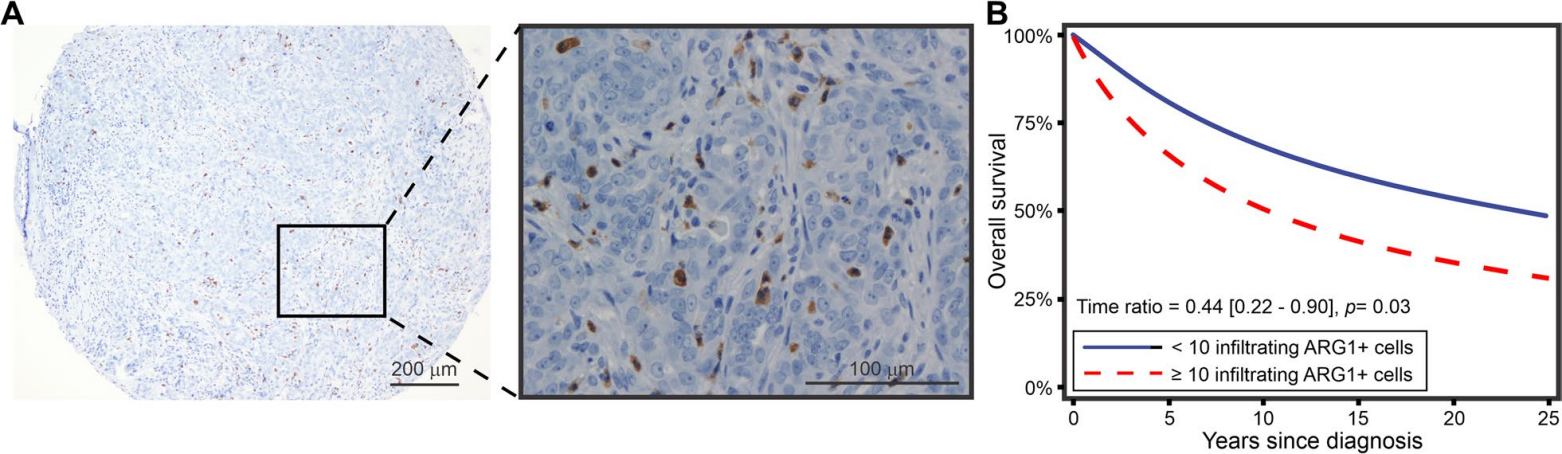
Immunohistochemistry of human breast cancer TMA (N = 487) and survival curve. **A** shows a representation of ARG1-positive cells' spatial distribution at 100X magnification. The picture to the right shows a zoom-in of the ARG1 positive staining at 400X magnification, which was observed as granular and cytoplasmic staining inside of polymorphonuclear cells. No cancer cells showed positive for ARG1 staining. **B** Survival curve analysis showing that a high infiltration of ARG1-positive cells was associated with lower breast cancer-specific survival. Patients were divided into two categories: < 10 and ≥ 10 ARG1 positively stained cells

ARG1-positivity was defined as granular cytoplasmic staining of ≥ 10 infiltrating polymorphonuclear cells per 1 mm tissue core and was observed in 33/487 (7%) cases. There was no evidence of ARG1 expression in epithelial tumour cells (Fig. 5A).

### ARG1 and histopathological grade and type

ARG1-positivity was associated with a high histopathological grade. Of the 33 ARG1-positive tumours, 2/33 (6%) were grade 1, 12/33 (36%) were grade 2, and 19/33 (58%) were grade 3 (p = 0.005). A greater proportion of ARG1-positive tumours was observed among special types of breast cancer than among ductal and lobular types (p = 0.03) (Table 1).

### ARG1 and molecular subtypes

ARG1-positive tumours (≥ 10 cells) were observed among all molecular subtypes. Most ARG1-positive cases were found among the luminal subtypes (12/259 (4%) luminal A, 5/115 (4%) luminal B (HER2^−^), and 2/35 (6%) luminal B (HER2^+^). However, the proportion of ARG1-positive cases was more significant among the non-luminal cases, where ARG1-positive cases were found in the HER2 type 6/27 (22%), 5NP 1/12 (8%), and BP 7/36 (19%) (p < 0.001) (Table 1).

### ARG1 and proliferation

The presence of 10 or more ARG1-positive cells was associated with increased proliferation. A total of 20/33 (61%) ARG1-positive tumours were found among the Ki67 high tumours (p = 0.04). ARG1-positive tumours were also found primarily in the upper quartiles of mitotic counts per high-power field, with 10/33 (30%) of the tumours having mitotic counts per HPF in the 3rd quartile and 13/33 (39%) with mitotic counts per HPF in the 4th quartile (p < 0.001) (Table 1).

### ARG1 and survival

The relative prognoses of cases with < 10 and ≥ 10 ARG1 positive cells were estimated using a log-normal accelerated failure time model. Women with ARG1-positive tumours had a significantly lower estimated survival time from diagnosis than women with ARG1-negative tumours (Fig. 5B). The time ratio for this variable was calculated to be 0.44 (95% CI 0.22–0.90), meaning that the average estimated lifespan of the ARG1-positive group from the time of diagnosis was only 44% of that of the ARG1-negative group. As shown in Additional file 1: Table S4, this time ratio remained significantly different from 1 when adjusted for age and histological subtype, but not when adjusted for grade, molecular subtype or Ki67 status.

## Discussion

Despite the critical role of myeloid cells in metastasis [8, 10, 11], their use as prognostic markers and therapeutic targets has been limited by their high level of heterogeneity. Hence, it is necessary to generate more insights into their functional diversity to understand how these cells contribute to metastasis. To address this, we compared non-metastatic 67NR and metastatic 66cl4 tumours from the immunocompetent 4T1 mouse breast cancer model, which enabled us to identify immune functions unique to the metastatic tumour. Our results show, for the first time, that ARG1 is predominantly expressed by myeloid cells in metastatic tumour but not in non-metastatic ones. Moreover, in human breast cancer patients a higher number of ARG1-positive infiltrating cells was associated with a high histopathological grade and proliferation.

We identified the ARG1-positive myeloid cells as Ly6G-intermediate immature neutrophils [30, 31] and monocytes [32]. It has been shown that immature neutrophils can infiltrate tumours [30, 33], and their levels in tumours correlate with tumour burden [30, 34]. However, Ly6G is also transiently expressed by monocytes during their development in the bone marrow [32], which can be recruited prematurely to the tumour [35]. Taking these results into account, we ascertain ARG1 is expressed by a heterogenous group of myeloid cells in metastatic 66cl4 tumours. Previously, ARG1 expression has been linked to tumour-associated myeloid cells, independently of the metastatic potential of the tumour [36–40]. Our work provided new insights by showing that ARG1-positive myeloid cells were abundant in metastatic tumours and rare in non-metastatic ones.

Interestingly, we found elevated levels of ARG1-positive myeloid cells predominantly inside the metastatic tumour, consistent with previous reports showing similar results in 4T1 [36] and PyMT-BO1 [40] metastatic breast tumours as well as colon carcinoma, lymphoma and melanoma [37] and glioma [41] mouse models. Since we found no immune cells with elevated ARG1 expression in the bone marrow or blood, we hypothesize that ARG1 expression was induced by local factors after tumour infiltration. This is in line with extensive evidence showing that intra-tumoral ARG1 expression can be induced by tumour secretome [41] especially; cytokines, growth factors, and hypoxia [40, 42]. Although cells positive for Arg1 mRNA and ARG1 protein predominantly localised in the tumour periphery, the deeper infiltration of ARG1 protein containing cells may suggest involvement of various cell types or cells of different maturation/activation stage. Further studies are needed to establish where, when and, in response to what signal the infiltrating myeloid cells induce the expression of ARG1.

We found a small number of ARG1 protein positive cells in the lungs of metastatic tumour-bearing mice, which could indicate early migration of these cells from the tumour to establish the pre-metastatic niche. Our results suggest that once Ly6G intermediate cells are released from the bone marrow or spleen, they rapidly migrate to the tumour where ARG1 expression is induced. Then, some ARG1-positive cells may leave the tumour and migrate to the lungs. It is well-known that neutrophils and other myeloid suppressive cells are essential for establishing metastatic niches [28, 43]. In addition, neutrophils can reach the tumour and return to circulation [44] through reverse migration, which has been proposed as a mechanism used by neutrophils to promote metastasis [45]. So, ARG1-positive cells observed in the lungs may be myeloid cells that migrate from the tumour, and they are presumably indicative for early steps in the metastatic process.

In humans, high ARG1 expression and activity have been reported in many cancers, often associating a high ARG1 expression with poor prognosis [46–49]. However, in those studies, high level of ARG1 was attributed to the whole tumours, requiring further investigations to determine ARG1-positive cell type and prognostic value of infiltrating ARG1-positive cells. To address the value of ARG1-positive cells as prognostic markers in breast cancer patients, we performed IHC on 487 biopsies. Our results showed that ARG1 protein was not expressed by cancer cells, but by cells with an appearance consistent with tumour-infiltrating immune cells. Additionally, while ARG1 positive cells were observed in all molecular subtypes, they were present at significantly higher levels in HER2 type and basal phenotype (BP), in line with our findings in the mouse preclinical model. HER2 [50] and BP [51] are fast-growing tumours that metastasize more readily than other tumour subtypes, suggesting an association between ARG1-positive cells and high grade and proliferation. Interestingly, a recent study investigating the presence of S100A8-positive tumour-infiltrating neutrophils in the same breast cancer patient cohort shows no association between a high number of S100A8-positive cells and unfavorable patient prognoses [52]. Together, these studies indicate that markers distinguishing immune cell populations alone may not be reliable markers for prognosis, while employing markers that reveal the biological function of these cells may be advantageous.

In our study, we used a homogeneous and well-described patient population, many of whom did not receive standard treatment for breast cancer because of the time or age at diagnosis. This allowed us to study this biomarker in the near-natural disease course after surgery. However, some limitations of this study include that only tissue samples from the tumour periphery were analysed, which could introduce a sample bias due to tumour heterogeneity. At the same time, the edge is the proliferative area of the tumour, and ARG1-positive cells are associated with proliferation. In addition, the patient group had a relatively high age at diagnosis. Since luminal tumours are more common among older women, the proportion of non-luminal tumour types is lower than expected in the population at large. Our results suggest that the infiltration of ARG1-positive cells is an independent prognostic biomarker for poor overall survival in human breast cancer.

## Conclusions

In our pursuit to understand the role of myeloid cells in metastatic breast cancer, we found an association between the presence of infiltrating ARG1-positive myeloid cells and aggressive tumour features in both breast cancer mouse models and patients. In addition, we demonstrated the translational potential of ARG1-positive cells as a prognostic marker for determining patient outcomes. Future research would focus on gaining deeper mechanistic insights into how metastatic tumours module myeloid cells' phenotype and how ARG1-positive myeloid cells influence tumour progression and response to anti-cancer therapies. Importantly, we would need to extend this study's results to a bigger cohort of cancer patients to validate ARG1-positive cells as a marker of an immunosuppressive tumour microenvironment. In the future, it could guide the clinical decision-making processes during cancer patients’ treatment and lead to the development of complementary therapies targeting the immunosuppressive function of myeloid cells.

### Supplementary Information

**Additional file 1:** **Figure S1.** **A** Principal component analysis (PCA) of the RNA-sequencing of CD45^+^ and CD45^−^ sorted cells from 67NR and 66cl4 tumours (N = 5). **B** Volcano plot of 67NR CD45^+^ cells vs 66cl4 CD45^+^ cells showing the differential genes expression (N = 5). **C** Gene ontology Molecular function analysis of the top 500 overexpressed genes in 66cl4 CD45^+^ indicates a significant association of these genes with CXC chemokine receptor activity and cytokines binding. **D** Gene ontology Cellular component analysis of the top 500 overexpressed genes in 66cl4 CD45^+^ shows a strong association of these genes with extracellular space and the external side of the plasma membrane. **Figure S2.** Arginase gene and protein expression at 2.5X and single cells at 400X. **A**–**D** show a representative image of in situ hybridisation (**A**, **B**) and immunohistochemistry (**C**, **D**). The spatial distribution of ARG1 gene and protein in 67NR and 66cl4 tumours is shown at 2.5X magnification. It shows the difference in ARG1 mRNA and protein abundance and distribution in 67NR and 66cl4 tumours. **E**, **F** show a representative image of ARG1 protein by IHC at 400X magnification, and the panel to the right shows a zoom-in of the ARG1-positive cell. **Figure S3.** Flow cytometry gating strategies on dissociated tumours and organs to identify ARG1-positive cells. To investigate the source of ARG1 in 67NR and 66cl4 tumours, antibodies mentioned in Materials and Methods were used. Forward/side angle light scatter (FSCA/SSC-A) was used to exclude debris, followed by a forward scatter height (FSC-H)/forward scatter area (FSC-A) to exclude doublets and Zombie Aqua (live dead marker)/FSC to exclude dead cells. **A** Gating strategy to identify ARG1-positive cells in immune and non-immune populations. The single and live cells were plotted for ARG1 vs CD45 (immune cell marker) to identify if immune (CD45^+^) or nonimmune (CD45^−^) cells were the primary source of ARG1. **B** Gating strategy to identify ARG1-positive immune cells. Single/live cells were plotted for CD45 vs FSC to distinguish between immune (CD45^+^) and non-immune (CD45^−^) cells. Further, the immune cells were plotted for ARG1 vs CD11b (myeloid cell marker) to identify if myeloid (CD11b^+^) or lymphoid (CD11b^-^) cells were the primary ARG1 source. FMOs were used to gate positive populations. **Figure S4.** Flow cytometry gating strategies on dissociated tumours/organs to identify immune subpopulations. Gating strategy to assess myeloid cell subpopulations in tumours. The resulting population of single, live cells were plotted for CD45 (immune cell marker) to distinguish between immune (CD45^+^) and non-immune (CD45^−^) cells. Immune cells were plotted for CD11b vs Ly6G (myeloid cell marker/neutrophil marker) to identify different immune cell populations. The plot revealed three distinct CD11b^+^ cell populations of interest: 1 subset of CD11b^+^/Ly6G^−^ and 2 population subsets of CD11b^+^/Ly6G^+^ cells. The two CD11b^+^/Ly6G^+^ cell populations could be distinguished based on the level of Ly6G as cells with intermediate or high Ly6G expression. These populations were labelled CD11b^+^/Ly6G^−^, CD11b^+^/Ly6G intermediate and CD11b^+^/Ly6G high. **Figure S5.** The frequencies of Ly6C^+^ vs F4/80^+^ cells in the 3 Ly6G populations: Ly6G^−^, LyG6^+^ intermediate, Ly6G^+^ high, in 67NR and 66cl4 tumours. Values are mean ± SEM of at least 3 independent experiments. **Figure S6.** Flow cytometry gating strategies on dissociated tumours and organs to identify ARG1-positive immune subpopulations. Gating approach to identify ARG1 source in CD11b^+^ subsets. Single, live cells were plotted for CD45 (immune cell marker), followed by CD11b (myeloid cell marker). The resulting myeloid cells were plotted for ARG1 vs Ly6G (neutrophils marker) to identify the CD45^+^/CD11b^+^ population source of ARG1. FMOs were used to gate positive populations. **Figure S7.** No elevated levels of ARG1-positive cells were found in blood, bone marrow and spleen in tumour-bearing mice. **A** The myeloid cell population (CD11b^+^) in blood, bone marrow, lung, and spleen have a similar composition based on Ly6G expression level in healthy and tumour-bearing animals. Blood (control mice N = 9, 67NR N = 9, 66cl4 N = 11), bone marrow (control mice N = 6, 67NR N = 6, 66cl4 N = 6), lungs (control mice N = 4, 67NR N = 4, 66cl4 N = 3) and spleen (control mice N = 3, 67NR N = 4, 66cl4 N = 3). Bars represent the mean ± SEM. Statistical analysis was performed using the Kruskal–Wallis test followed by Dunn’s multiple comparisons (ns, p > 0.05; *p < 0.05). **B** Representative flow cytometry plots representing ARG1-positive cells in immune cells (CD45^+^) from blood, bone marrow, lungs, and spleen of healthy and tumour-bearing mice. **Figure S8.** ARG1 protein quantification in plasma by ELISA. Bars represent mean ± SEM, and each data point represents a single animal (control N = 10, 67NR N = 14; 66cl4 N = 14). Statistical significance was determined using one-way ANOVA followed by Kruskal–Wallis test multiple comparisons. **p < 0.005. **Table S1.** Gene Ontology analysis of Biological Process elevated in 66cl4 CD45^+^ cells compared with 67NR CD45^+^ cells. **Table S2.** Fn1, Tnbs1 and Arg1 expression levels as transcripts per million in the CD45^+^ and CD45^-^ cells isolated from 67NR and 66cl4 tumours. **Table S3.** Frequencies of ARG1-positive cells in the blood, bone marrow, lungs and spleen. **Table S4.** Parameter estimates for the log-normal accelerated failure time model, unadjusted and adjusted for other variables.

## Data Availability

The RNA sequencing data are scheduled to be publicly available from February 01, 2024; in the Gene Expression Omnibus (GEO) at GSE211223. The data generated in this study are available from the corresponding authors upon request.

## Abbreviations

5NP: 5 Negative Phenotype
Alox15: Arachidonate 15-lipoxygenase
ARG1: Arginase 1
BP: Basal Phenotypes
CK5: Cytokeratin 5
CXCL3: Chemokine (C–X–C motif) ligand 3
DMEM: Dulbecco's Modified Eagle Medium
EGFR: Epidermal Growth Factor Receptor
ELISA: Enzyme Linked Immunosorbent Assay
ER: Estrogen Receptor
F7: Coagulation factor VII
FCS: Fetal Calf Serum
Fn1: Fibronectin 1
Foxf1: Forkhead Box F1
GO: Gene Ontology
HER2: Human Epidermal growth factor Receptor 2
HRP: Horse Radish Peroxidase
ICB: Immune Checkpoint Blockade
IHC: Immunohistochemistry
Il1a: Interleukin 1 alpha
Il23: Interleukin 23
ISH: In Situ Hybridization
MDSC: Myeloid Derived Suppressor Cells
Nt5e: 5′-Nucleotidase or ecto-5′-nucleotidase
PMN: Polymorphonuclear cells
PR: Progesterone Receptor
Ptgis: Prostaglandin I2 Synthase
RBC: Red Blood Cell
Scara5: Scavenger receptor class A member 5
Serpinb2: Serpin Family B Member 2
Thbs1: Thrombospondin 1
TMA: Tissue Microarrays
TME: Tumour Microenvironment
WBC: White blood cells
