# Supplementary material for: Discovery of a new marker to identify myeloid cells associated with metastatic breast tumours
Source: Cancer Cell Int. 2023 Nov 18;23:279. doi: 10.1186/s12935-023-03136-w (PMC10656772; doi:10.1186/s12935-023-03136-w)
Supplement: Supplementary file 1 — Additional file 1: Figure S1. A Principal component analysis (PCA) of the RNA-sequencing of CD45+ and CD45− sorted cells from 67NR and 66cl4 tumours (N = 5). B Volcano plot of 67NR CD45+ cells vs 66cl4 CD45+ cells showing the differential genes expression (N = 5). C Gene ontology Molecular function analysis of the top 500 overexpressed genes in 66cl4 CD45+ indicates a significant association of these genes with CXC chemokine receptor activity and cytokines binding. D Gene ontology Cellular component analysis of the top 500 overexpressed genes in 66cl4 CD45+ shows a strong association of these genes with extracellular space and the external side of the plasma membrane. Figure S2. Arginase gene and protein expression at 2.5X and single cells at 400X. A–D show a representative image of in situ hybridisation (A, B) and immunohistochemistry (C, D). The spatial distribution of ARG1 gene and protein in 67NR and 66cl4 tumours is shown at 2.5X magnification. It shows the difference in ARG1 mRNA and protein abundance and distribution in 67NR and 66cl4 tumours. E, F show a representative image of ARG1 protein by IHC at 400X magnification, and the panel to the right shows a zoom-in of the ARG1-positive cell. Figure S3. Flow cytometry gating strategies on dissociated tumours and organs to identify ARG1-positive cells. To investigate the source of ARG1 in 67NR and 66cl4 tumours, antibodies mentioned in Materials and Methods were used. Forward/side angle light scatter (FSCA/SSC-A) was used to exclude debris, followed by a forward scatter height (FSC-H)/forward scatter area (FSC-A) to exclude doublets and Zombie Aqua (live dead marker)/FSC to exclude dead cells. A Gating strategy to identify ARG1-positive cells in immune and non-immune populations. The single and live cells were plotted for ARG1 vs CD45 (immune cell marker) to identify if immune (CD45+) or nonimmune (CD45−) cells were the primary source of ARG1. B Gating strategy to identify ARG1-positive immune cells. Single/live [file 12935_2023_3136_MOESM1_ESM.pdf]

Additional files

FIGURES

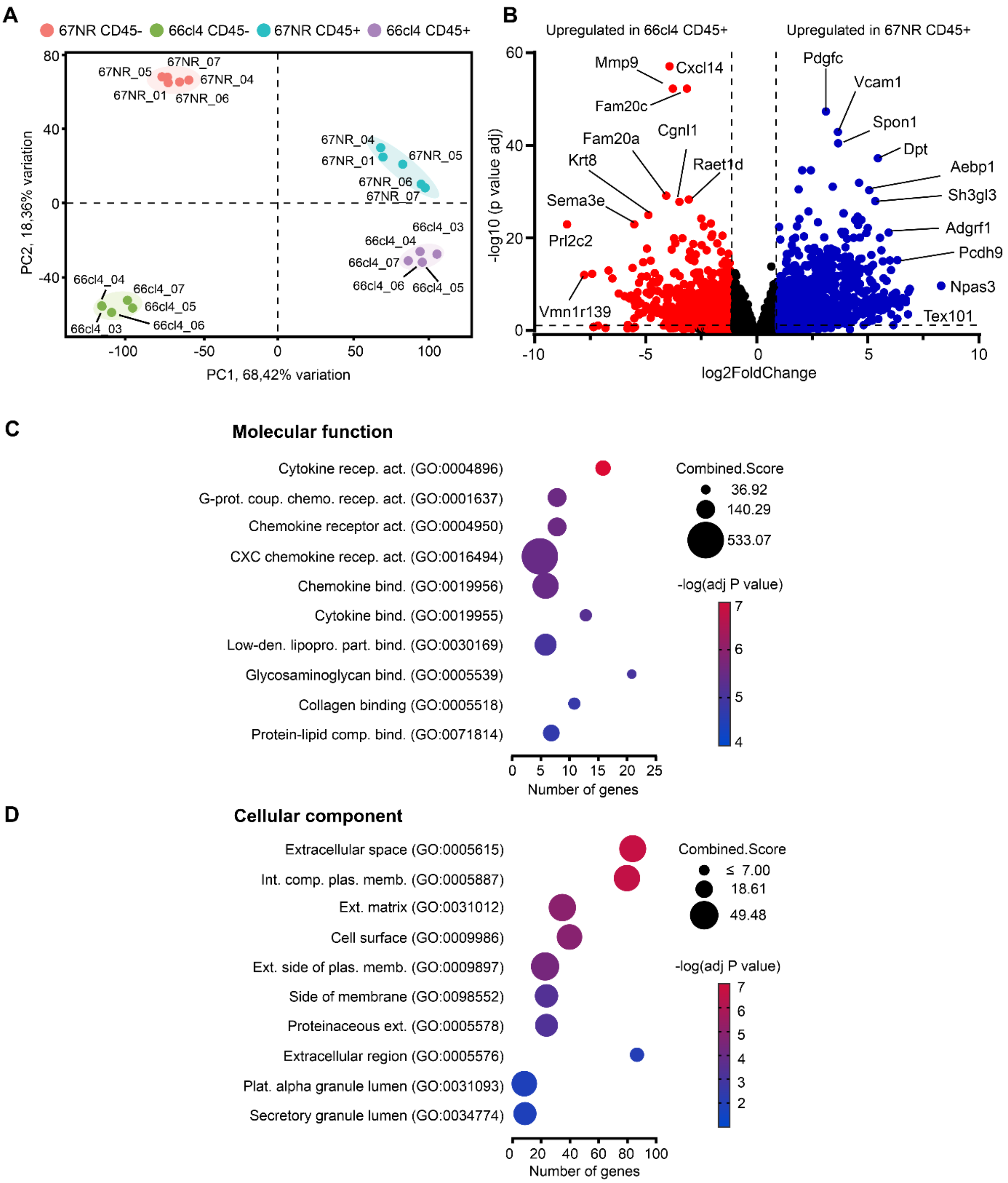

Figure S1: A) Principal component analysis (PCA) of the RNA-sequencing of CD45+ and CD45- sorted cells from 67NR and 66cl4 tumours (n=5). B) Volcano plot of 67NR CD45+ cells vs 66cl4 CD45+ cells showing the differential genes expression (n=5). C) Gene ontology Molecular function analysis of the top 500 overexpressed genes in 66cl4 CD45+ indicates a significant association of these genes with CXC chemokine receptor activity and cytokines binding. D) Gene ontology Cellular

component analysis of the top 500 overexpressed genes in 66cl4 CD45+ shows a strong association of these genes with extracellular space and the external side of the plasma membrane.

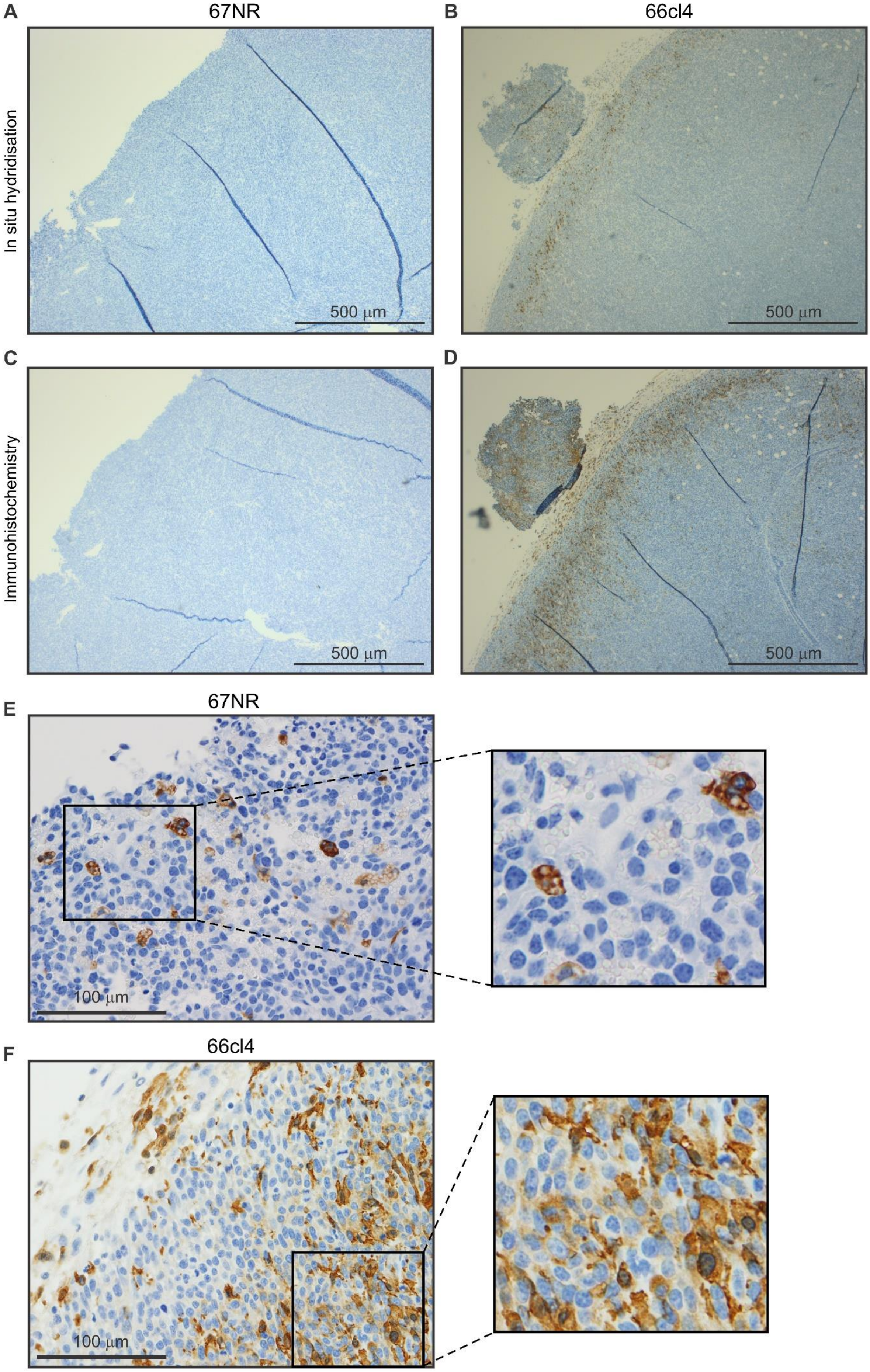

A
67NR
B
66cl4
In situ hydridisation
500 µm
500 µm
C
D
Immunohistochemistry
500 µm
500 µm
E
67NR
100 µm
F
66cl4
100 µm

Figure S2: Arginase gene and protein expression at 2.5X and single cells at 400X. Panels A-D show a representative image of in situ hybridisation (A-B) and immunohistochemistry (C-D). The spatial distribution of ARG1 gene and protein in 67NR and 66cl4 tumours is shown at 2.5X magnification. It shows the difference in ARG1 mRNA and protein abundance and distribution in 67NR and 66cl4 tumours. Panels E-F show a representative image of ARG1 protein by IHC at 400X magnification, and the panel to the right shows a zoom-in of the ARG1 positive cell.

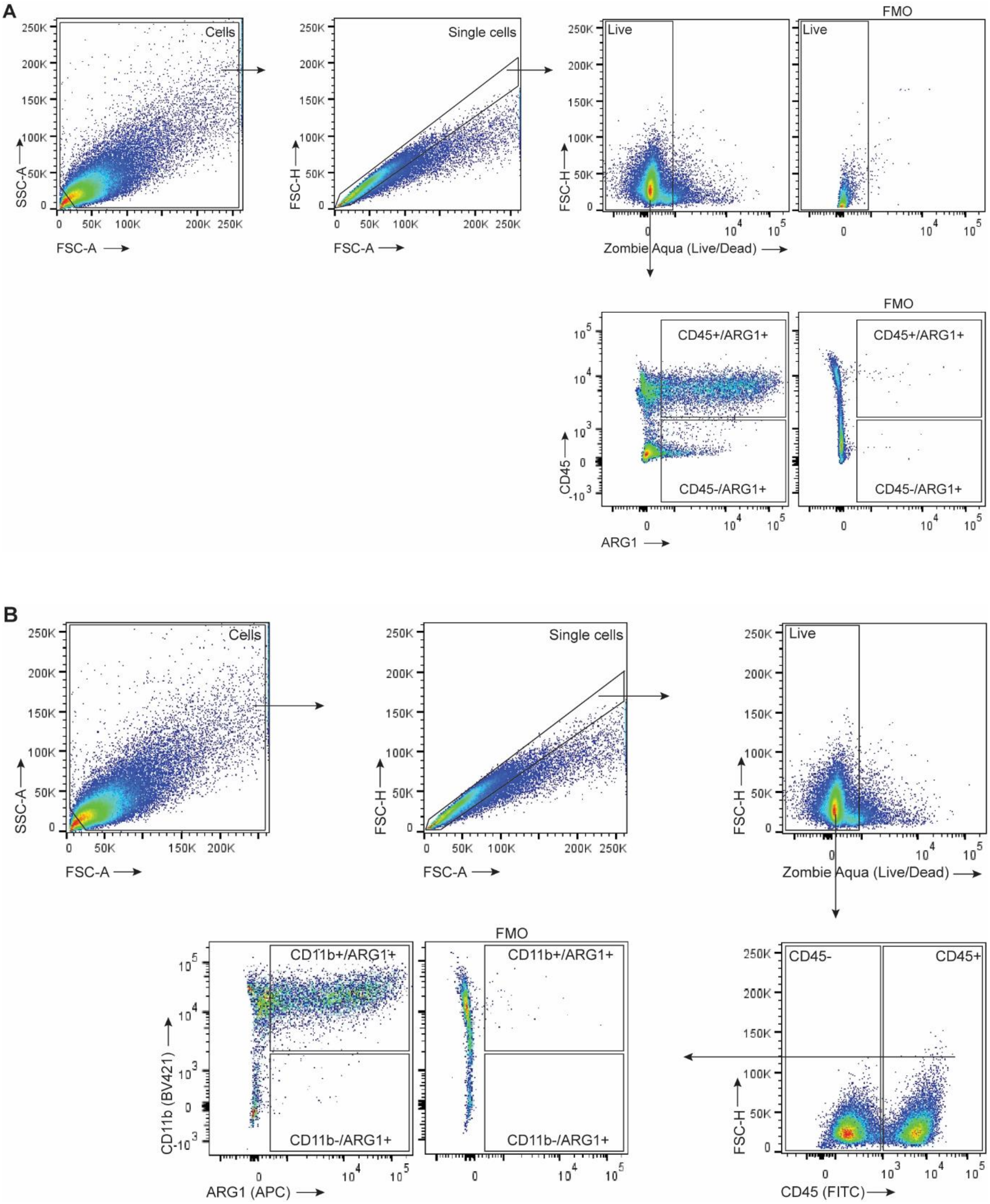

Figure S3 shows flow cytometry gating strategies on dissociated tumours and organs to identify ARG1-positive cells. To investigate the source of ARG1 in 67NR and 66cl4 tumours, antibodies mentioned in Materials and Methods were used. Forward/ side angle light scatter (FSC-A/SSC-A) was used to exclude debris, followed by a forward scatter height (FSC-H)/ forward scatter area (FSC-A) to exclude doublets and Zombie Aqua (live dead marker)/ FSC to exclude dead cells. A) Gating strategy to identify ARG1 positive cells in immune and non-immune populations. The single and live cells were plotted for ARG1 vs CD45 (immune cell marker) to identify if immune (CD45+) or non-immune (CD45-) cells were the primary source of ARG1. B) Gating strategy to identify ARG1-positive

immune cells. Single/live cells were plotted for CD45 vs FSC to distinguish between immune (CD45+) and non-immune (CD45-) cells. Further, the immune cells were plotted for ARG1 vs CD11b (myeloid cell marker) to identify if myeloid (CD11b+) or lymphoid (CD11b-) cells were the primary ARG1 source. FMOs were used to gate positive populations.

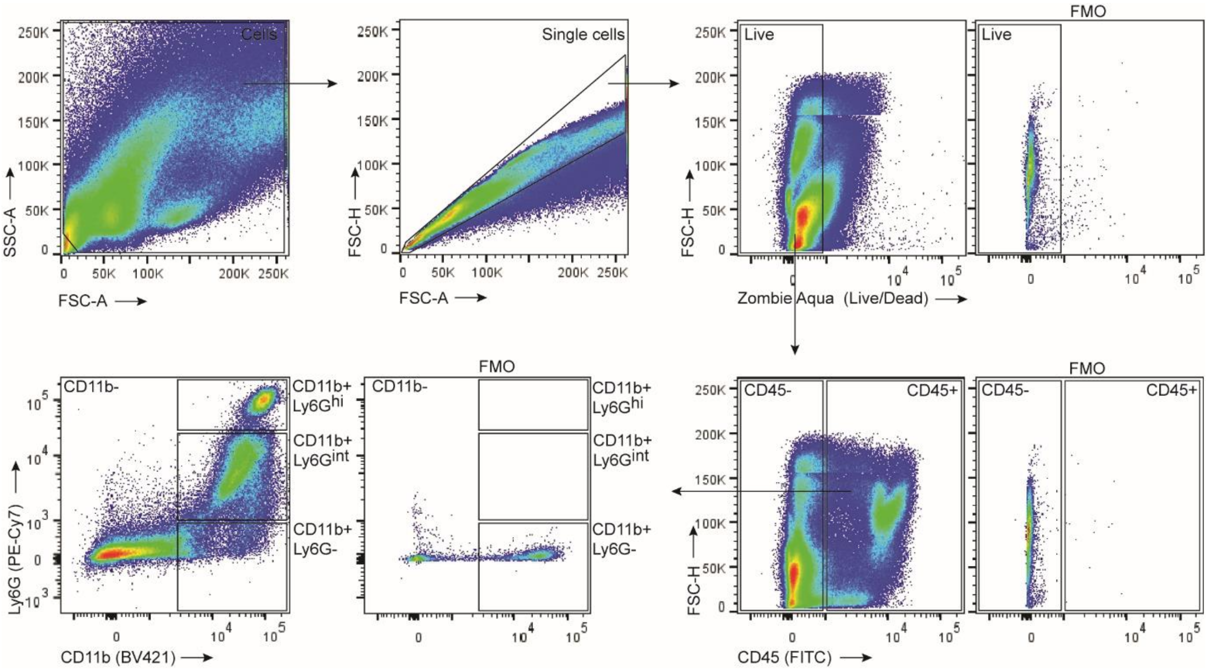

Figure S4 shows flow cytometry gating strategies on dissociated tumours/organs to identify immune subpopulations. Gating strategy to assess myeloid cell subpopulations in tumours. The resulting population of single, live cells were plotted for CD45 (immune cell marker) to distinguish between immune (CD45+) and non-immune (CD45-) cells. Immune cells were plotted for CD11b vs Ly6G (myeloid cell marker/neutrophil marker) to identify different immune cell populations. The plot revealed three distinct CD11b+ cell populations of interest: 1 subset of CD11b+/ Ly6G- and 2 population subsets of CD11b+/Ly6G+ cells. The two CD11b+/Ly6G+ cell populations could be distinguished based on the level of Ly6G as cells with intermediate or high Ly6G expression. These populations were labelled CD11b+/Ly6G-, CD11b+/ Ly6G intermediate and CD11b+/Ly6G high.

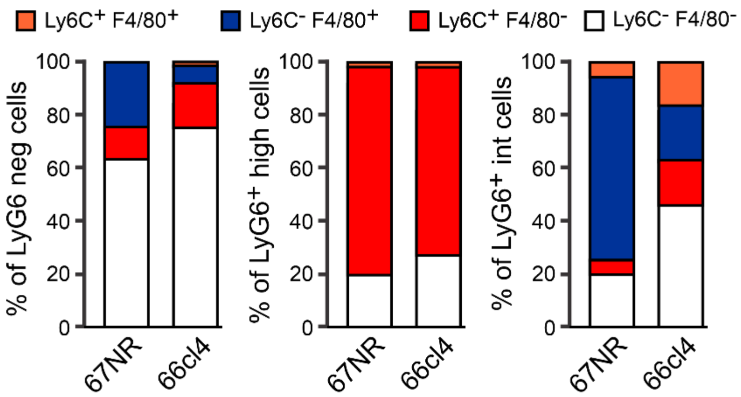

Figure S5 shows the frequencies of Ly6C+ vs F4/80+ cells in the 3 Ly6G populations: Ly6G-, LyG6+ intermediate, Ly6G+ high, in 67NR and 66cl4 tumors. Values are means ± SEM of at least 3 independent experiments.

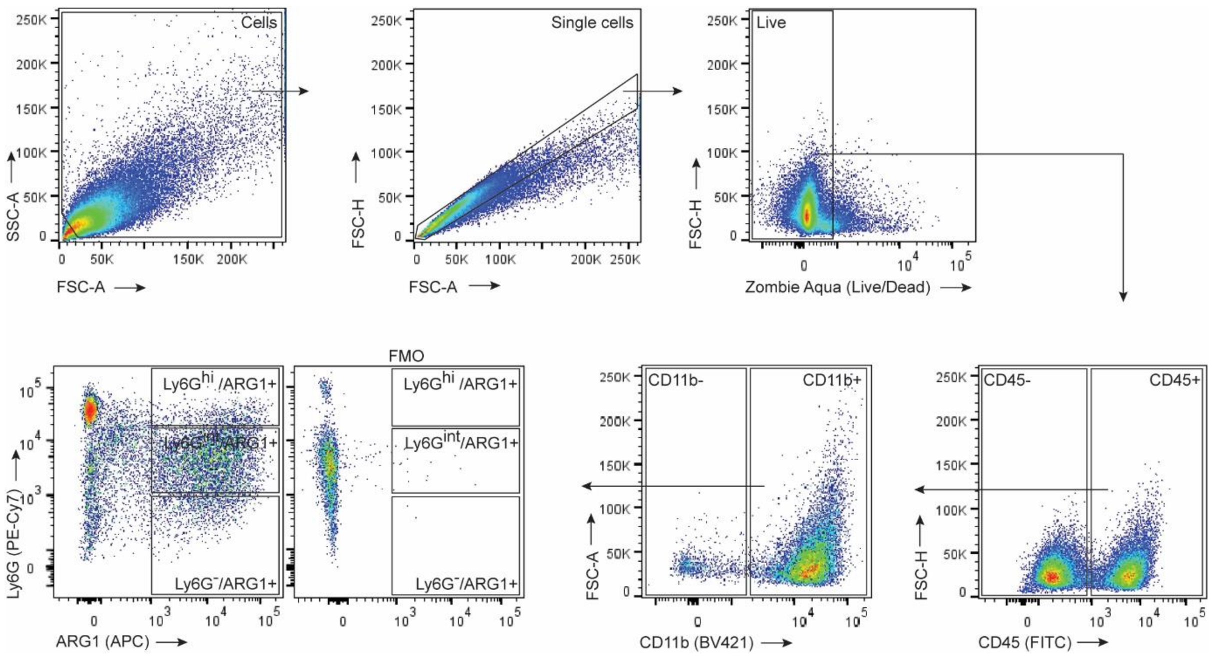

Figure S6 shows flow cytometry gating strategies on dissociated tumours and organs to identify ARG1+ immune subpopulations. Gating approach to identify ARG1 source in CD11b+ subsets. Single, live cells were plotted for CD45 (immune cell marker), followed by CD11b (myeloid cell marker). The resulting myeloid cells were plotted for ARG1 vs Ly6G (neutrophils marker) to identify the CD45+/CD11b+ population source of ARG1. FMOs were used to gate positive populations.

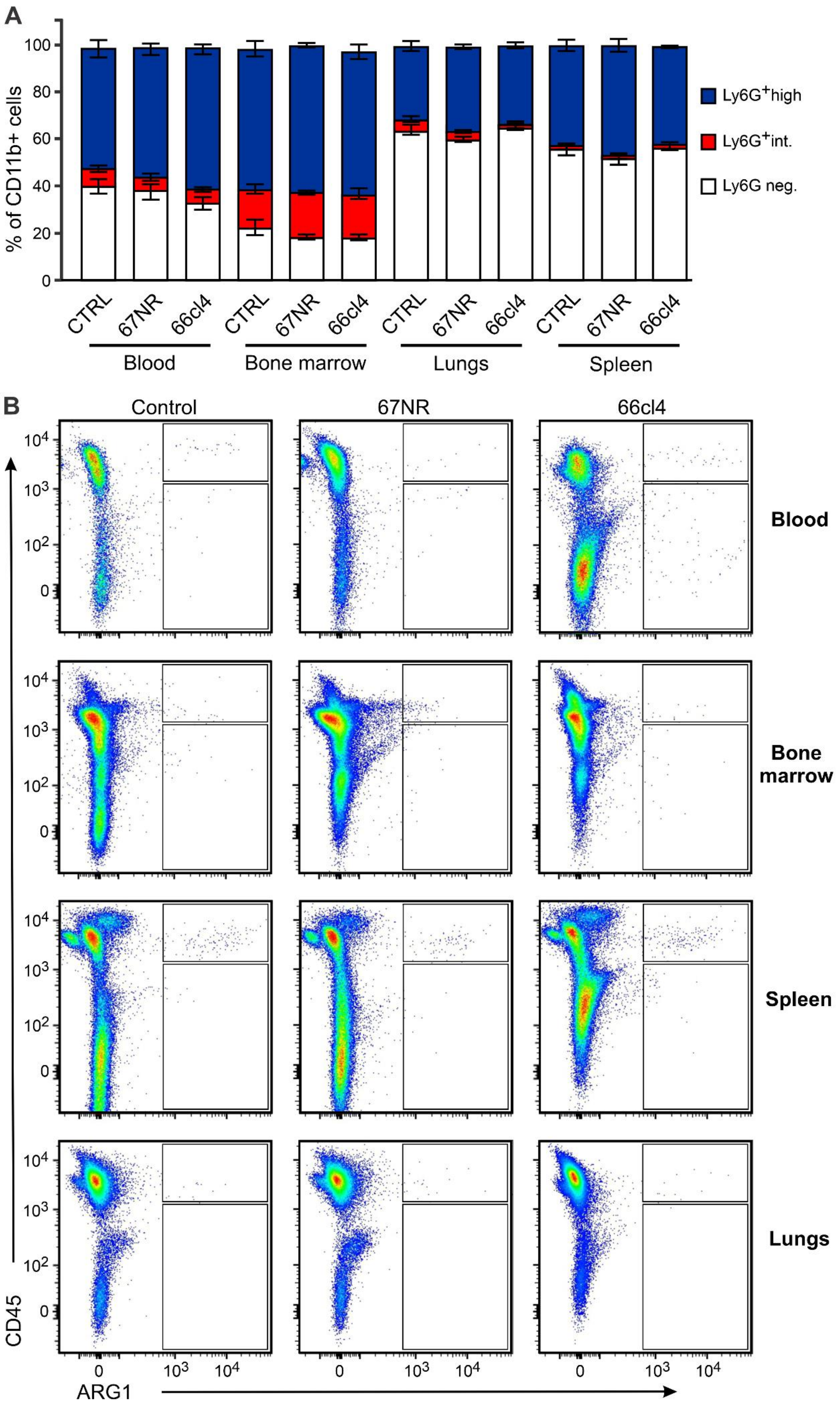
A
% of CD11b+ cells
0
20
40
60
80
100
Ly6G+high
Ly6G+int.
Ly6G neg.
CTRL
67NR
66cl4
Blood
Bone marrow
Lungs
Spleen
B
Control
67NR
66cl4
Blood
Bone marrow
Spleen
Lungs
CD45
ARG1

Figure S7 shows no elevated levels of ARG1-positive cells were found in blood, bone marrow and spleen in tumour-bearing mice. (A) The myeloid cell population (CD11b+) in blood, bone marrow, lung, and spleen have a similar composition based on Ly6G expression level in healthy and tumour-bearing animals. Blood (Control mice N=:9, 67NR N=9, 66cl4 N=11), bone marrow (Control mice N=6, 67NR N=6, 66cl4 N=6), lungs (Control mice N=4, 67NR N=4, 66cl4 N=3) and spleen (Control mice N=3, 67NR N=4, 66cl4 N=3). Bars represent the means ± SEM. Statistical analysis was performed using the Kruskal-Wallis test followed by Dunn's multiple comparisons (ns, $p>0.05$; *, $p<0.05$). (B) Representative flow cytometry plots representing ARG1+ cells in immune cells (CD45+) from blood, bone marrow, lungs, and spleen of healthy and tumour-bearing mice.

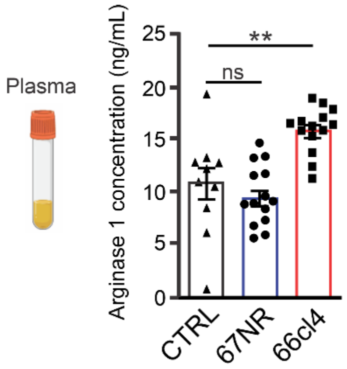

Figure S8 shows ARG1 protein quantification in plasma by ELISA. Bars represent means ± SEM, and each data point represents a single animal (Control n: 10, 67NR n:14; 66cl4 n:14). Statistical significance was determined using one-way ANOVA followed by Kruskal-Wallis test multiple comparisons. ** p<0.005.

TABLES

Table S1 Gene Ontology analysis of Biological Process elevated in 66cl4 CD45+ cells compared with 67NR CD45+ cells.

Term	Overlap	Adjusted P.value	Odds Ratio	Combined Score	Genes
leukocyte migration (GO:0050900)	35/226	4.341003841 26102e-09	4.849403 7230948 2	133.26742 7025314	RET;CSF3R;C5AR1;CXCR4;SLC7A11 ;TREM1;CXCR1;DPYSL3;CXCR2;SP P1;PDE4B;S1PR1;OLR1;CCR7;CCR6 ;LBP;SLC16A3;JAM2;CCL24;TGFB2; CXADR;SPNS2;FN1;L1CAM;MMP9;V EGFA;SELP;NLRP12;COL1A2;SELL; F2RL1;ADAM8;S100A9;CD44;S100A8
taxis (GO:0042330)	37/263	6.378146633 4997e-09	4.336671 4324450 9	112.74397 9096565	CSF3R;C5AR2;SEMA3A;C5AR1;FPR 1;CXCR5;CXCR4;FPR2;CXCL14;CXC L2;TREM1;CXCR1;FLRT3;DPYSL3;C XCR2;SPP1;PDE4B;S1PR1;CCR7;CC R6;LBP;CCL24;TGFB2;CXADR;EGR3 ;PLAUR;VEGFC;L1CAM;VEGFA;BMP 4;NR4A1;ACKR4;ACKR3;ADAM8;PLP 2;S100A9;S100A8
chemotaxis (GO:0006935)	37/263	6.378146633 4997e-09	4.336671 4324450 9	112.74397 9096565	CSF3R;C5AR2;SEMA3A;C5AR1;FPR 1;CXCR5;CXCR4;FPR2;CXCL14;CXC L2;TREM1;CXCR1;FLRT3;DPYSL3;C XCR2;SPP1;PDE4B;S1PR1;CCR7;CC R6;LBP;CCL24;TGFB2;CXADR;EGR3 ;PLAUR;VEGFC;L1CAM;VEGFA;BMP 4;NR4A1;ACKR4;ACKR3;ADAM8;PLP 2;S100A9;S100A8
ext. matrix organization (GO:0030198)	43/359	1.859848663 32882e-08	3.617670 8505191 3	88.662705 4391107	ECM2;OLFML2B;COL14A1;ITGB4;CO L12A1;LTBP4;TNFRSF11B;NDNF;TH BS1;COMP;ADAMTS5;ADAMTS2;CT SL;CTSK;SPP1;COL10A1;DMD;MPZL 3;JAM2;TGFB2;COL27A1;LAMB3;FO XF1;FN1;KAZALD1;MMP9;MMP12;B MP4;GREM1;VCAN;COL1A2;P4HA1; ELF3;CRISPLD2;ITGA10;COL4A6;IT GA8;SDC1;MMP19;ADAM8;COL6A6; CD44;PLEC
ext. structure organization (GO:0043062)	43/360	1.859848663 32882e-08	3.606068 1246234	88.048594 2066088	ECM2;OLFML2B;COL14A1;ITGB4;CO L12A1;LTBP4;TNFRSF11B;NDNF;TH BS1;COMP;ADAMTS5;ADAMTS2;CT SL;CTSK;SPP1;COL10A1;DMD;MPZL 3;JAM2;TGFB2;COL27A1;LAMB3;FO XF1;FN1;KAZALD1;MMP9;MMP12;B MP4;GREM1;VCAN;COL1A2;P4HA1; ELF3;CRISPLD2;ITGA10;COL4A6;IT GA8;SDC1;MMP19;ADAM8;COL6A6; CD44;PLEC

inflammatory response (GO:0006954)	42/376	2.19873039441218e-07	3.33524535781173	72.5899292825413	HDAC5;IL1RN;C5AR2;C5AR1;ALOX15;HP;CXCR4;IL5RA;FPR2;MEFV;PTGS2;CXCL2;THBS1;CXCR1;CNR2;IRAK2;GPER1;CXCR2;SPP1;OLR1;NLRP3;CCR7;S1PR3;CD14;LBP;KDM6B;CCL24;SLC11A1;FN1;SELP;IL1A;ELF3;IL23A;TNFSF4;TNIP3;SDC1;F2RL1;ADAM8;TLR6;TLR5;S100A9;S100A8
pos. reg. of resp. to wounding (GO:1903036)	22/130	1.7996492227827e-06	5.31825072002425	103.748685369461	CCL24;NTRK3;OSM;PTGS2;F3;THBS1;HOPX;IL17RA;SELP;F7;NLRP12;IL23A;ADORA2B;TNFSF4;ADAM8;CCR7;LBP;CD36;S100A9;S100A8;TGM2;NDEL1
reg. of res. to wounding (GO:1903034)	38/347	2.00310900631753e-06	3.24783686070602	62.5773945642739	MEFV;PTGS2;METRNL;KLK8;THBS1;SLC7A2;NT5E;CNR2;GPER1;SPP1;NLRP3;CCR7;CD36;LBP;IER3;NDEL1;TGM2;CCL24;SERPINB2;PTGIS;SCARA5;FOXF1;NTRK3;OSM;PLAUR;F3;HOPX;IL17RA;SELP;F7;NLRP12;IL23A;ADORA2B;TNFSF4;F2RL1;ADAM8;S100A9;S100A8
leukocyte chemotaxis (GO:0030595)	19/100	2.26710967143739e-06	6.10768921095008	116.203787373924	CCL24;TGFB2;CSF3R;CXADR;C5AR1;CXCR4;TREM1;VEGFA;CXCR1;DPYSL3;CXCR2;PDE4B;SPP1;ADAM8;CCR7;LBP;CCR6;S100A9;S100A8
pos. reg. of cell migration (GO:0030335)	33/280	2.76344191848661e-06	3.51550461493602	65.8189891694938	RET;CEMIP;IRS1;SEMA3A;IRS2;PTGS2;HIF1A;THBS1;IGF1R;WNT11;GPER1;GCNT2;S1PR1;CCR7;LBP;CCL24;TGFB2;F10;FOXF1;ANXA3;NTRK3;VEGFC;F3;MMP9;VEGFA;SELP;BMP4;FGR;F7;IL23A;F2RL1;CDH13;ADAM8
resp. to mol. of bact. origin (GO:0002237)	30/243	4.15386917071892e-06	3.69732880038854	67.3639016680598	IL1RN;CEBPE;PTGER2;C5AR1;PALM3;PTGS2;LITAF;CXCL2;CNR2;IRAK2;DPYSL3;CD300LB;PDE4B;NLRP3;CCR7;CD14;CD36;LBP;ABCA1;PTGIR;ARG1;SLC11A1;SELP;TNFSF4;TNIP3;TLR6;TRIB1;TLR5;S100A9;S100A8
pos. reg. of cell motility (GO:2000147)	33/287	4.25623486482536e-06	3.41736100508212	61.8824527181152	RET;CEMIP;IRS1;SEMA3A;IRS2;PTGS2;HIF1A;THBS1;IGF1R;WNT11;GPER1;GCNT2;S1PR1;CCR7;LBP;CCL24;TGFB2;F10;FOXF1;ANXA3;NTRK3;VEGFC;F3;MMP9;VEGFA;SELP;BMP4;FGR;F7;IL23A;F2RL1;CDH13;ADAM8
cell chemotaxis (GO:0060326)	23/155	5.74645293008196e-06	4.54958395429707	80.6551009897733	CCL24;TGFB2;CSF3R;CXADR;EGR3;C5AR1;CXCR4;CXCL14;CXCL2;TREM1;VEGFA;NR4A1;CXCR1;DPYSL3;CXCR2;PDE4B;SPP1;ADAM8;CCR7;LBP;CCR6;S100A9;S100A8
pos. reg. of cell. comp. mov. (GO:0051272)	33/296	7.78075426656074e-06	3.29885299600813	57.2378651563382	RET;CEMIP;IRS1;SEMA3A;IRS2;PTGS2;HIF1A;THBS1;IGF1R;WNT11;GPER1;GCNT2;S1PR1;CCR7;LBP;CCL24;TGFB2;F10;FOXF1;ANXA3;NTRK3;VEGFC;F3;MMP9;VEGFA;SELP;BMP4;FGR;F7;IL23A;F2RL1;CDH13;ADAM8

resp. to LPS (GO:0032496)	28/228	1.0441003869927e-05	3.66753782668501	62.3032452477223	IL1RN;CEBPE;PTGER2;C5AR1;PALM3;PTGS2;LITAF;CNR2;IRAK2;DPYSL3;CD300LB;PDE4B;NLRP3;CCR7;CD14;CD36;LBP;ABCA1;PTGIR;ARG1;SLC11A1;SELP;TNFSF4;TNIP3;TRIB1;TLR5;S100A9;S100A8

Table S2: Fn1, Tnbs1 and Arg1 expression levels as transcripts per million in the CD45+ and CD45- cells isolated from 67NR and 66cl4 tumours.

**Gene**	**67NR**				**66cl4**			
	**CD45-**		**CD45+**		**CD45-**		**CD45+**	
	**Mean**	**SEM**	**Mean**	**SEM**	**Mean**	**SEM**	**Mean**	**SEM**
**Fn1**	96721	10804	15906	1212	77553	11635	131562	43173
**Thbs1**	2483	298	5955	1422	32171	1538	41968	1512
**Arg1**	83	66	980	426	133	52	13633	2498

Table S3: Frequencies of ARG1+ cells in the blood, bone marrow, lungs and spleen

Organ	*Mouse*	*CD45- (% ARG1+ cells)*				*CD45+ (% ARG1+ cells)*			
		Mean	SEM	n	p-value	Mean	SEM	n	p-value
Blood	*Control*	0,074	0,017	7		0,204	0,063	7	
	67NR	0,043	0,012	6	ns	0,382	0,167	6	ns
	66cl4	0,070	0,025	6	ns	0,396	0,174	6	ns
Bone marrow	*Control*	0,036	0,008	4		0,045	0,022	4	
	67NR	0,020	0,010	4	ns	0,021	0,004	4	ns
	66cl4	0,020	0,002	3	ns	0,035	0,014	3	ns
Lung	*Control*	0,036	0,012	4		0,170	0,013	4	
	67NR	0,033	0,010	4	ns	0,119	0,025	4	ns
	66cl4	0,079	0,036	3	ns	0,227	0,029	3	$^{\#}$
Spleen	*Control*	0,016	0,008	4		0,056	0,018	4	
	67NR	0,005	0,001	4	ns	0,026	0,007	4	ns
	66cl4	0,004	0,001	3	ns	0,019	0,005	3	ns

Statistical test: Kruskal-Wallis test followed by Dunn's multiple comparisons (*, $p < 0.05$ compared with Control; $^{\#}$, $p < 0.05$ compared with 67NR.

Table S4: Parameter estimates for the log-normal accelerated failure time model, unadjusted and adjusted for other variables.

	Infiltration of ≥ 10 ARG1+ cells	
	No	Yes
TR unadjusted (95 % CI)	1	0.44 (0.22 - 0.90)
TR unadjusted for age (95 % CI)	1	0.44 (0.22 - 0.88)
TR unadjusted for the grade (95 % CI)	1	0.56 (0.28 - 1.13)
TR unadjusted for histology subtype (95 % CI)	1	0.42 (0.20 - 0.86)
TR unadjusted for molecular subtype (95 % CI)	1	0.65 (0.32 - 1.32)
TR unadjusted for Ki67 (95 % CI)	1	0.54 (0.27 - 1.1)

TR time ratio, CI confidence interval.
